# A highly adaptive microbiome-based association test for survival traits

**DOI:** 10.1186/s12864-018-4599-8

**Published:** 2018-03-20

**Authors:** Hyunwook Koh, Alexandra E. Livanos, Martin J. Blaser, Huilin Li

**Affiliations:** 1 Department of Population Health, New York University School of Medicine, 650 First Avenue, Room 547, New York, NY 10016 USA; 2 Department of Medicine, Columbia University Medical Center, New York, NY 10032 USA; 3 Departments of Medicine and Microbiology, New York University School of Medicine, New York, NY 10016 USA; 4 Medical Service, New York Harbor Department of Veterans Affairs Medical Center, New York, NY 10010 USA

**Keywords:** Microbiome-based survival analysis, Microbiome-based association test, Community-level association test, Microbial group analysis, High-dimensional compositional data analysis, Phylogenetic tree

## Abstract

**Background:**

There has been increasing interest in discovering microbial taxa that are associated with human health or disease, gathering momentum through the advances in next-generation sequencing technologies. Investigators have also increasingly employed prospective study designs to survey survival (i.e., time-to-event) outcomes, but current item-by-item statistical methods have limitations due to the unknown true association pattern. Here, we propose a new adaptive microbiome-based association test for survival outcomes, namely, optimal microbiome-based survival analysis (OMiSA). OMiSA approximates to the most powerful association test in two domains: 1) microbiome-based survival analysis using linear and non-linear bases of OTUs (MiSALN) which weighs rare, mid-abundant, and abundant OTUs, respectively, and 2) microbiome regression-based kernel association test for survival traits (MiRKAT-S) which incorporates different distance metrics (e.g., unique fraction (UniFrac) distance and Bray-Curtis dissimilarity), respectively.

**Results:**

We illustrate that OMiSA powerfully discovers microbial taxa whether their underlying associated lineages are rare or abundant and phylogenetically related or not. OMiSA is a semi-parametric method based on a variance-component score test and a re-sampling method; hence, it is free from any distributional assumption on the effect of microbial composition and advantageous to robustly control type I error rates. Our extensive simulations demonstrate the highly robust performance of OMiSA. We also present the use of OMiSA with real data applications.

**Conclusions:**

OMiSA is attractive in practice as the true association pattern is unpredictable in advance and, for survival outcomes, no adaptive microbiome-based association test is currently available.

**Electronic supplementary material:**

The online version of this article (10.1186/s12864-018-4599-8) contains supplementary material, which is available to authorized users.

## Background

The human microbiota is the totality of all microorganisms living in and on the human body [1] and its role in human health and disease has been increasingly studied [2–5]. Human microbiota studies have been accelerated by the advent of next-generation sequencing technologies which enabled an unbiased characterization of all microorganisms, often by targeting the bacterial 16S ribosomal RNA (rRNA) gene [6, 7]. Diverse microorganisms can be identified based on sequence similarity to known 16S rRNA genes and classified into operational taxonomic units (OTUs) [8]. The OTUs are characterized by their estimated abundance (e.g., read count or relative abundance) and phylogenetic tree structure (i.e., taxonomical and evolutionary relationships). Accordingly, various microbial diversity metrics on the basis of microbial abundance and phylogenetic tree information have been surveyed in microbiome-based association studies [9]. The data are also large-scale including numerous OTUs (e.g., hundreds to thousands) with the presence of a long tail of rare microorganisms. We now pursue further discovery of associated microbial taxa for more integrative assessments about the root causes of maladies.

Recently, a number of microbiome-based association tests have been introduced to survey the entire microbial community (e.g., bacterial kingdom) and microbial taxa (e.g., phyla, classes, orders, families, genera, and species). As statistical power from massive univariate analyses for individual OTUs is considerably low due to the requisite multiple testing correction, here, our focus lies on association tests for microbial groups of multiple OTUs (i.e., the entire community (e.g., bacterial kingdom) and higher-level taxa (e.g., phyla, classes, orders, families, and genera)). A majority of existing methods (e.g., LEfSe [10], STAMP [11], DESeq2 [12], and metagenomeSeq-fit Zig [13]) relate aggregated microbial abundance for each taxon with health or disease outcome [14]. However, these methods are subject to a substantial loss of power as its underlying assumption - the same effect direction for all associated OTUs - is violated (e.g., within a taxon of interest, some OTUs are symbiotic, while others are pathogenic) [14]. To explain, when OTUs in a taxon of interest are all positively (or all negatively) associated with an outcome of interest (i.e., in the case of the same effect direction), their positive (or negative) association signals are amplified in their aggregated abundance, so that we can powerfully discover the association between the aggregated abundance and the outcome of interest. However, when OTUs in a taxon of interest are in mixed effect directions (i.e., some are positively, while others are negatively associated with an outcome of interest), their positive and negative association signals are canceled out in their aggregated abundance, so that we cannot discover any (positive or negative) association between the aggregated abundance and the outcome of interest. Detailed description and simulation studies have been addressed in [14]. Moreover, those aggregate-based methods do not utilize phylogenetic tree structure which considers taxonomical and evolutionary relationships among diverse microorganisms. As an alternative, distance-based analysis is popular and, for example, microbiome regression-based kernel association test (MiRKAT) [15] is spotlighted in this context. MiRKAT incorporates diverse ecologically informative distance metrics (e.g., unique fraction (UniFrac) distance [16–18] and Bray-Curtis dissimilarity [19]) into its kernel machine regression framework. As different distance metrics vary in the extent of the relative contributions from microbial abundance and phylogenetic tree information, they can be most accurate in different true underlying association patterns, respectively. However, prior knowledge about the true association pattern is limited and it is thus difficult to predict which distance metric is optimal in practice. The adaptive test of MiRKAT, called Optimal MiRKAT, approximates to an optimal test adaptively among multiple MiRKAT tests using different distance metric specifications; hence, in practice, Optimal MiRKAT is attractive. OMiAT [14] is a further adaptive test which approximates to an optimal test adaptively throughout the sum of powered score tests (SPU) [20] and MiRKAT tests. By including SPU tests in the search space, OMiAT robustly discovers rare, mid-abundant, and abundant associated lineages along with the functionality of Optimal MiRKAT.

There has also been increasing interest in discovering microbial taxa that are associated with survival (i.e., time-to-event) outcomes on the basis of prospective study designs (e.g., randomized clinical trials and prospective cohort studies) [21–23]. Survival outcomes are better informed by examining health or disease progression at multiple times over a lengthy period of follow-up. However, all of the above methods can handle only binary or continuous outcomes at a single time point. Currently, a remarkable association testing method in microbiome-based survival analysis is microbiome regression-based kernel association test for survival traits (MiRKAT-S) [24]. As with MiRKAT, MiRKAT-S incorporates distance metrics into its kernel machine regression framework, but is designed to handle survival outcomes. Plantinga et al. [24] also demonstrated that MiRKAT-S has higher power than other distance-based approaches used in prior studies, such as Cox proportional hazards regression followed by principal coordinates analysis [21] or Ward’s agglomerative hierarchical clustering method [25].

However, MiRKAT-S has three critical issues. First, Plantinga et al. [24] reports that MiRKAT-S performs poorly when associated OTUs are rare in abundance. Microbiome data usually contain mostly rare OTUs and only few OTUs representing most of the abundance, especially for gut or oral microbiota which has greater microbial diversity. This indicates that if the test works only for few dominant associated OTUs, numerous rare or mid-abundant taxa are simply ignored. As a remedy, we introduce a new set of association tests, namely, microbiome-based survival analysis using linear and non-linear bases of OTUs (MiSALN), which weigh rare, mid-abundant, and abundant OTUs, respectively, and its adaptive testing method, Optimal MiSALN (OMiSALN), to ensure a robust performance for OTUs with low or high abundance. Second, MiRKAT-S handles distance metrics one-by-one and no adaptive testing method is available. The cherry-picking approach from multiple item-by-item MiRKAT-S tests cannot correctly control type I error rate or the requisite multiple testing correction can lead to a substantial loss of power. Therefore, we also introduce an adaptive testing method for MiRKAT-S, namely, Optimal MiRKAT-S (OMiRKAT-S). Third, as with MiRKAT, MiRKAT-S can assess only the entire community and is not currently applicable to higher-level taxa. Thus, we extend its usability as a general microbial group analytic method.

Our major proposed method is a highly adaptive test, namely, optimal microbiome-based survival analysis (OMiSA), which creates an optimal test throughout multiple MiSALN and MiRKAT-S tests. As a result, OMiSA performs well whether the underlying associated lineages are rare or abundant by MiSALN and phylogenetically related or not by MiRKAT-S.

We now present the methodological details of the approach, and then provide extensive simulations and real data applications, before discussing limitations and other feasibilities.

## Methods

**Fig. 1:**
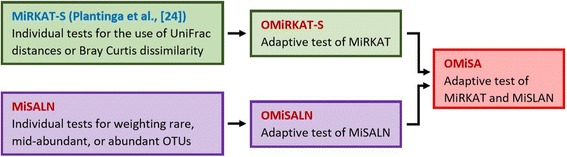
An overview which organizes existing methods (blue letters) and our new methods (red letters). Our major proposed method is OMiSA

This section is devoted to describe the methodological details of our proposed methods. Here, we first organize our new methods separated from existing methods in Fig. 1. That is, MiRKAT-S [24] for the individual use of different distance metrics is an existing method (blue letters, Fig. 1), and our methods (red letters, Fig. 1) are its adaptive test, OMiRKAT-S, MiSALN and its adaptive test, OMiSALN, and OMiSA. Again, OMiSA is our major proposed method, and the other individual and sub-adaptive tests are necessary to reach our final destination, OMiSA.

### Models and notations

Suppose that there are n subjects, p OTUs, and q covariates (e.g., age and sex) and the subscripts, i, j, and k, indicate a subject, an OTU, and a covariate, respectively. For each subject i, let T_i_ be a survival time, C_i_ be a censoring time, and Y_i_ be an observed time. Then, Y_i_ is defined as Y_i_ = min(T_i_, C_i_) and an event indicator, δ_i_, is defined as δ_i_ = I(T_i_ ≤ C_i_). The ordered observed event times are denoted by τ_1_, ..., τ_m_, where m is the number of total events (m ≤ n), and the risk set at time τ_g_, is denoted by R_g_, for g = 1, ..., m. In addition, for each subject i, denote a p × 1 vector, Z_i_, for the microbial composition of the entire community or a higher-level taxon, marked for its elements in OTU-level relative abundance as Z_ij_ for j = 1, ..., p, and denote a k × 1 vector, X_i_, for the covariates, marked for its elements as X_ik_ for k = 1, ..., q. Here, we assume that n subjects are identically and independently distributed (e.g., random subjects) and C_i_ is independent of T_i_ conditional on Z_i_ and X_i_.

To relate microbial composition with survival outcomes adjusting for covariates, we consider a Cox proportional hazard model (Eq. 1) [26].

1 $$ {\uplambda}_{\mathrm{i}}\left(\mathrm{t}\right)={\uplambda}_0\left(\mathrm{t}\right){\mathrm{e}}^{\Sigma_{\mathrm{k}=1}^{\mathrm{q}}{\upalpha}_{\mathrm{k}}{\mathrm{X}}_{\mathrm{i}\mathrm{k}}+\mathrm{h}\left({\mathrm{Z}}_{\mathrm{i}}\right)}, $$

where λ_i_(t) is the conditional hazard function given Z_i_ and X_i_, λ_0_(t) is the baseline hazard function, α’s are coefficients for the effect of covariates (e.g., age and sex), and h(Z_i_) is a function which characterizes the relationship between microbial composition and survival outcomes. For example, if we specify h(Z_i_) = $\sum \limits_{\mathrm{j}=1}^{\mathrm{p}}{\upbeta}_{\mathrm{j}}{\mathrm{Z}}_{\mathrm{ij}}$, we can relate the linear effects of OTUs in relative abundance to the log hazard rate. The non-linear effects of OTUs in relative abundance can also be surveyed by the use of non-linear bases of OTUs (e.g., polynomials/splines) [27]. Moreover, we can specify h(Z_i_) more flexibly by the use of different positive semi-definite kernel functions modeled for different distance/similarity metrics among subjects [27, 28]. In the following sections, we introduce two different machineries for the specification of h(Z_i_) - one for the use of different linear/non-linear bases of OTUs and the other for the use of different kernel functions - and illustrate how their performance varies by different true underlying association patterns. The former is a newly introduced method, MiSALN. The latter is an existing method, MiRKAT-S [24]; hence, we describe its main ideas and formula and refer to its original paper for more details. Of most importance is that we introduce new adaptive testing methods which approximate to an optimal test for each of the two machineries (namely, OMiSALN and OMiRKAT-S, respectively) and throughout the two different machineries (namely, OMiSA).

### MiSALN

Suppose that β = (β_1_, …, β_p_)^T^ is the vector of regression coefficients for p OTUs. We are particularly interested in testing the null hypothesis of no association between the microbial composition consisting of those p OTUs and survival outcomes, H_0_: β = (β_1_, …, β_p_)^T^ = 0. Assuming the coefficients, β_1_, ..., β_p_, are random and independent with mean zero, a common variance, σ^2^, and a pairwise correlation matrix, Ρ, (i.e., E(β) = 0 and Cov(β) = σ^2^Ρ), the same null hypothesis and its corresponding alternative can be formulated as a variance-component score test with Eq. 2 [28–32].

2 $$ {\mathrm{H}}_0:{\upsigma}^2=0\;\mathrm{vs}.{\mathrm{H}}_1:{\upsigma}^2>0 $$

Note here that any full distributional form for the coefficients, β_1_, …, β_p_, was not required, but we need to specify the correlation matrix, Ρ [29]. For the choice of Ρ, we can consider different choices which have greater deviations from H_0_ in directions corresponding to the larger eigenvalues of P [29]. However, we consider the p × p identity matrix, I_p_, for no correlation over β_j_’s for MiSALN [30].

The Cox proportional hazard model for the null hypothesis can be formulated with Eq. 3.

3 $$ {\uplambda}_{\mathrm{i}}\left(\mathrm{t}\right)={\uplambda}_0\left(\mathrm{t}\right){\mathrm{e}}^{\Sigma_{\mathrm{k}=1}^{\mathrm{q}}}{\upalpha}_{\mathrm{k}}{\mathrm{X}}_{\mathrm{i}\mathrm{k}} $$

Based on the maximum likelihood estimates, $\widehat{\upalpha}$’s, the estimated cumulative hazard rate for subject i at its observed time, ${\widehat{\Lambda}}_{\mathrm{i}}$, can be derived as in Eq. 4 [29]. We here used Efron’s approximation [33] to handle observations which have tied survival times.

4 $$ {\widehat{\Lambda}}_{\mathrm{i}}={\Sigma}_{\uptau_g\le {\mathrm{Y}}_{\mathrm{i}}}\frac{\exp \left({\Sigma}_{\mathrm{k}=1}^{\mathrm{q}}{\widehat{\upalpha}}_{\mathrm{k}}{\mathrm{X}}_{\mathrm{i}\mathrm{k}}\right)}{\Sigma_{\mathrm{f}\in {\mathrm{R}}_{\mathrm{g}}}\exp \left({\Sigma}_{\mathrm{k}=1}^{\mathrm{q}}{\widehat{\upalpha}}_{\mathrm{k}}{\mathrm{X}}_{\mathrm{f}\mathrm{k}}\right)} $$

Based on $\widehat{\upalpha}$’s and the resulting estimates, ${\widehat{\Lambda}}_{\mathrm{i}}$’s, Verweij et al. [29] derives a variance-component score test statistic to test H_0_: σ^2^ = 0 against H_1_: σ^2^ > 0 as in Eq. 5.

5 $$ \mathrm{U}={\left(\mathrm{d}-\hat{\Lambda}\right)}^{\mathrm{T}}\mathrm{R}\left(\mathrm{d}-\hat{\Lambda}\right), $$

where d = (δ_1_, …, δ_n_)^T^, $\widehat{\mathrm{e}}$ = ${\left({\widehat{\Lambda}}_1,\dots, {\widehat{\Lambda}}_{\mathrm{n}}\right)}^{\mathrm{T}}$, and R is the n × n correlation matrix for n subjects that we need to specify. Here, $\mathrm{d}-\widehat{\Lambda}$ is the vector of the estimated martingale residuals under the null model (Eq. 3). Verweij et al. also derives the mean and variance of U and demonstrates that the null distribution of the standardized score test closely approximates to standard normal distribution [29]. However, since our proposed tests are based on a residual permutation-based scheme for *p*-value calculation and the mean and variance of U are evaluated under the null, the unstandardized score test, U, is sufficient in our study.

Of importance is that with different specifications for R, we can survey different correlation structures among subjects. For MiSALN, our choices for R are formulated with Eq. 6.

6 $$ {\mathrm{R}}_{\mathrm{MiSALN}\left(\upgamma \right)}={\mathrm{Z}}^{\upgamma}{\mathrm{Z}}^{\upgamma^{\mathrm{T}}}, $$

where Z is the n × p matrix for OTU relative abundances (i.e., compositions), Z = (Z_1_, …, Z_n_)^T^, and γ (ϵ *ℝ*^+^) powers Z and needs to be pre-specified. The variance-component score test with this correlation matrix, R_MiSALN(γ)_, can be simply derived as in Eq. 7.

7 $$ {\mathrm{U}}_{\mathrm{MiSALN}\left(\upgamma \right)}={\left(\mathrm{d}-\hat{\Lambda}\right)}^{\mathrm{T}}{\mathrm{R}}_{\mathrm{MiSALN}\left(\upgamma \right)}\left(\mathrm{d}-\hat{\Lambda}\right), $$

The correlation structure, R_MiSALN(γ)_, describes pairwise similarities in microbial abundance among subjects and the variance-component score test, U_MiSALN(γ)_, represents the degree of overall association between R_MiSALN(γ)_ and the estimated martingale residuals [30, 31]. Here, only the microbial abundance information is contributed and no phylogenetic tree information is incorporated.

Note that, γ transforms OTUs to the γ’s power of the original relative abundances, so that different bases of OTUs can be surveyed. When γ = 1, the original scale of OTUs is used for testing the linear effect of OTUs. The resulting correlation matrix, ZZ^T^, is equivalent to the linear kernel in kernel machine regression models [28] and has also been used for a gene-set association testing method, namely, Global Test [30]. When γ ≠ 1, the non-linear bases of OTUs can be surveyed. Here, we demonstrate different γ value specifications as different weighting schemes for OTUs in relative abundance as follows. As γ increases, abundant OTUs will be relatively weighted, while rare OTUs will gradually be lost, but vice versa as γ decreases. Therefore, we can expect that when abundant OTUs are associated with survival outcomes, a large value of γ can be more suitable by weighting them more, but vice versa when rare OTUs are associated. However, in practice, the true underlying association pattern is mostly unknown and we cannot presume whether rare, mid-abundant, or abundant OTUs are associated with survival outcomes. Therefore, we propose a data-driven approach, Optimal MiSALN (OMiSALN), which approximates to an optimal test adaptively through different γ value specifications and its test statistic is formulated with Eq. 8.

8 $$ {\mathrm{Q}}_{\mathrm{OMiSALN}}=\underset{\gamma \in \Gamma}{\min }{\mathrm{P}}_{\mathrm{MiSALN}\left(\upgamma \right)}, $$

where Г is a set of candidate γ values and P_MiSALN(γ)_ is the estimated *p*-value for MiSALN(γ), where γϵГ. We can observe that Q_OMiSALN_ is the minimum *p*-value among different MiSALN(γ) tests, where γϵГ. Again, Q_OMiSALN_ itself is a test statistic which requires its own *p*-value estimation. We use a residual-based permutation method to estimate *p*-values for individual MiSALN(γ) tests, where γϵГ, and OMiSALN (see Additional file 1).

For a set of candidate γ values, we used Г = {1/4, 1/3, 1/2, 1} and it was sufficient in our simulations and real data analysis.

### MiRKAT-S

The key idea behind MiRKAT-S [24] is that diverse distance metrics (e.g., UniFrac distance [16–18] and Bray-Curtis dissimilarity [19]) can be incorporated into the kernel machine Cox proportional hazards model. Hence, we can survey the relationship between ecologically related metrics and survival outcomes on health or disease with covariate adjustments (e.g., age and sex) [24]. First, we need to specify a sample-by-sample pairwise distance matrix based on a chosen distance metric and transform it into a kernel (similarity) matrix using the kernel formula, Eq. 9.

9 $$ \mathrm{K}=-\frac{1}{2}\left(\mathrm{I}-\frac{11^{\hbox{'}}}{\mathrm{n}}\right){\mathrm{D}}^2\left(\mathrm{I}-\frac{11^{\hbox{'}}}{\mathrm{n}}\right), $$

where D is the n × n pairwise distance matrix and D^2^ is its element-wise square, I is the n × n identity matrix, and 1 in 11′ is the vector of n ones. To ensure the kernel matrix, K, to be positive semi-definite, negative eigenvalues are replaced with zero [24]. Then, using the resulting kernel matrix, the variance-component score statistic can be formulated with Eq. 10 [24, 27].

10 $$ {\mathrm{U}}_{\mathrm{MiRKAT}-\mathrm{S}\left(\mathrm{k}\right)}={\left(\mathrm{d}-\hat{\Lambda}\right)}^{\mathrm{T}}{\mathrm{K}}_{\left(\mathrm{k}\right)}\left(\mathrm{d}-\hat{\Lambda}\right), $$

where k is an index for a particular kernel matrix based on a chosen distance metric. Plantinga et al. [24] has also proposed a modified score statistic which accounts for over-dispersion, but since we calculate *p*-values based on a residual permutation-based method and the dispersion parameter, $\frac{1}{{\left(\mathrm{d}-\widehat{\Lambda}\right)}^{\mathrm{T}}\left(\mathrm{d}-\widehat{\Lambda}\right)}$, is evaluated under the null, the variance-component score test statistic of Eq. 10 is sufficient in our study.

Importantly, different distance metrics reflect different relative contributions from microbial abundance and phylogenetic tree information; as such, the performance of MiRKAT-S differs according to the choice of distance metric and the true underlying association pattern [16–18, 24]. The UniFrac distances are constructed based on phylogenetic tree information and the contribution of microbial abundance is modulated by different weighting schemes. The unweighted UniFrac distance incorporates only microbial presence/absence information so that it is most inclined to phylogenetic tree information [16], whereas the weighted UniFrac distance further incorporates microbial abundances [17]. In this context, the generalized UniFrac distance is regarded as a compromised version between the unweighted and weighted UniFrac distances [18]. In contrast, the Bray-Curtis dissimilarity [19] does not incorporate any phylogenetic tree information so that it is most inclined to microbial abundance information. Accordingly, when associated OTUs are phylogenetically related, the UniFrac distances can be better choices than Bray-Curtis dissimilarity, but vice versa when they are not phylogenetically related. However, we cannot predict which distance metric is optimal to our study. Therefore, here, we proposed a data-driven approach, namely, Optimal MiRKAT-S (OMiRKAT-S), which is taken adaptively through multiple distance metric specifications and its test statistic is formulated with Eq. 11.

11 $$ {\mathrm{Q}}_{\mathrm{OMiRKAT}-\mathrm{S}}=\underset{\mathrm{k}\in \uppsi}{\min }{\mathrm{P}}_{\mathrm{MiRKAT}-\mathrm{S}\left(\mathrm{k}\right),} $$

where Ψ is a set of candidate distance metrics and P_MiRKAT − S(k)_ is the estimated *p*-value for U_MiRKAT − S(k)_, where kϵΨ. Note that, OMiRKAT-S is similar to Optimal MiRKAT [15], but the difference is that OMiRKAT-S handles survival outcomes, while Optimal MiRKAT handles binary or continuous outcomes at a time point. Here again, Q_OMiRKAT − S_ is the minimum *p*-value among different MiRKAT-S(k) tests, where kϵΨ, and it is a test statistic that requires its own *p*-value estimation. Similar to MiSALN(γ)/OMiSALN, a residual-based permutation method was used to estimate *p*-values for individual MiRKAT-S(k) tests, where kϵΨ, and OMiRKAT-S (see Additional file 1).

For a set of candidate distance metrics, Ψ, we used Ψ = {unweighted UniFrac (K_U_), generalized UniFrac(0.5) (K_0.5_), weighted UniFrac (K_W_), Bray-Curtis (K_BC_)}, where K_0.5_ is the generalized UniFrac distance with the parameter, ϴ = 0.5, as suggested [9].

### OMiSA

Our major proposed method, OMiSA, approximates to an optimal test adaptively throughout all the different variance-component score tests of MiSALN(γ), where γϵГ, and MiRKAT-S(k), where kϵΨ, and its test statistic can be simply formulated with Eq. 12.

12 $$ {\mathrm{Q}}_{\mathrm{OMiSA}}=\min \left({\mathrm{Q}}_{\mathrm{OMiSA}\mathrm{LN}},{\mathrm{Q}}_{\mathrm{OMiRKAT}-\mathrm{S}}\right) $$

Q_OMiSA_ is the minimum *p*-value among different MiSALN(γ) tests, where γϵГ, and MiRKAT-S(k) tests, where kϵΨ. It is a test statistic, like Q_OMiSALN_ (Eq. 8) and Q_OMiRKAT − S_ (Eq. 11). We do not report this genuine minimum *p*-value as the final *p*-value to be reported for OMiSA, but estimate its *p*-value using a residual permutation-based method (see Additional file 1). We emphasize that throughout the machineries of MiSALN and MiRKAT-S, OMiSA powerfully discovers microbial taxa whenever their underlying associated OTUs are rare or abundant by MiSALN and phylogenetically related or not by MiRKAT-S. Our extensive simulations in later sections also demonstrate the robust performance of OMiSA.

### Assessment of higher-level taxa

We extend all the individual and adaptive tests as general group analytic methods which can assess any higher-level taxon (e.g., phyla, classes, orders, families, and genera), not only the entire community (e.g., bacterial kingdom), as long as they include multiple OTUs with phylogenetic tree structure. The only matter that requires attention is that when we assess higher-level taxa, their OTU relative abundances (i.e., compositions) are computed based on total reads in the entire community (i.e., OTU relative abundances are not sub-compositions which have unit sum to each higher-level taxon). Specifically, such normalization needs to be applied to the OTU relative abundances for MiSALN and to the UniFrac distances for MiRKAT-S.

### A graphical representation

As for visual representations of discoveries, we used an existing software tool, GraPhlAn [34], which is addressed later in our real data analysis. As GraPhlAn is flexibly customizable with beautiful circular representations of hierarchical taxonomic tree [34], here, we do not introduce any new graphical representation and suggest to use GraPhlAn after obtaining outcomes from OMiSA.

## Results

### Simulations

This section is devoted to simulations which evaluate individual MiSALN and MiRKAT-S tests and their adaptive tests, OMiSALN, OMiRKAT-S, and OMiSA in terms of type I error and statistical power. While the association testing methods can be applied to higher-level taxa, as a demonstration, here, we survey the entire community.

### Simulation design

We simulated microbiome data according to prior approaches [35] which are based on a Dirichlet-multinomial distribution reflecting real microbial composition. We first estimated proportion means and a dispersion parameter to be inserted into the Dirichlet-multinomial distribution using actual intestinal microbiome data of non-obese diabetic (NOD) mice in [23]. The complete microbiome data include NOD mice in different treatment groups and sequencing time points; however, as a demonstration, we selected 35 fecal samples from NOD mice at 6 weeks of age in the control group which had not been disturbed by antibiotic exposure. Then, 353 OTUs which have proportional mean abundance > 10^−4^ were included in the analysis. The total reads per sample was set to be 1000 [15, 24]. Based on these specifications, we simulated OTU counts for small (n = 50) and large (n = 100) samples, respectively, from the Dirichlet-multinomial distribution.

Two covariates for age and sex were simulated from a normal distribution, N(50, 5^2^) and a Bernoulli distribution, Bern(0.5), respectively. The survival time, T_i_, was simulated through Eq. 13, assuming proportional hazards and a Weibull distribution, Weibull(2,2), for the baseline at age = 50 and sex = 0 [28, 36].

13 $$ {\mathrm{T}}_{\mathrm{i}}=\sqrt{-\frac{4\log {\mathrm{U}}_{\mathrm{i}}}{\exp \operatorname{}\Big(0.5\left({\mathrm{age}}_{\mathrm{i}}-50\right)+0.5{\mathrm{sex}}_{\mathrm{i}}+{\Sigma}_{\mathrm{j}=1}^{\mathrm{p}}{\beta}_{\mathrm{j}}\mathrm{scale}\left({\mathrm{Z}}_{\mathrm{i}\mathrm{j}}\right)}} $$

where U_i_ was randomly sampled from a uniform distribution, Unif(0,1), *β*_j_ is a coefficient for each OTU j = 1,…,p, and scale(Z_ij_) = $\frac{{\mathrm{Z}}_{\mathrm{ij}}-\mathrm{mean}\left({\mathrm{Z}}_{1\mathrm{j}},{\mathrm{Z}}_{2\mathrm{j}},\dots, {\mathrm{Z}}_{\mathrm{nj}}\right)}{\mathrm{SD}\left({\mathrm{Z}}_{1\mathrm{j}},{\mathrm{Z}}_{2\mathrm{j}},\dots, {\mathrm{Z}}_{\mathrm{nj}}\right)}$, for subjects *i* = 1, …, n and OTUs j = 1, …, p. The censoring time, C_i_, was simulated based on uniform distribution with two different schemes to survey different extent of censoring: 1) C_i_ ~ Unif(0,10) which is of the estimated proportions of censored outcomes, 25.78%, and 25.88%, for small (n = 50) and large samples (n = 100), respectively, and 2) C_i_ ~ Unif(0,5) which is of the estimated proportions of censored outcomes, 40.42% and 40.48%, for small (n = 50) and large samples (n = 100), respectively (Table 1). Then, the observed time, Y_i_, and the event indicator, δ_i_, were calculated by the formula, Y_i_ = min(T_i_, C_i_) and δ_i_ = I(T_i_ ≤ C_i_), respectively.

**Table 1:** Estimated type I errors and proportions of censored outcomes for different sample sizes and censoring schemes (Unit: %)

Method	Small samples (n = 50)		Large samples (n = 100)	
	Unif(0,10)	Unif(0,5)	Unif(0,10)	Unif(0,5)
MiRKAT-S(K_U_)	4.97	4.99	4.94	4.99
MiRKAT-S(K_0.5_)	4.99	4.98	5.10	5.03
MiRKAT-S(K_W_)	5.00	4.92	5.09	5.06
MiRKAT-S(K_BC_)	4.98	4.93	5.00	4.99
OMiRKAT-S	4.97	4.91	5.06	5.12
MiSALN(1/4)	4.99	4.96	4.91	5.13
MiSALN(1/3)	5.04	4.95	4.93	5.13
MiSALN(1/2)	4.98	4.92	4.88	5.06
MiSALN(1)	4.96	4.93	4.90	5.03
OMiSALN	5.01	4.93	4.92	5.07
OMiSA	4.94	4.94	4.98	5.15
Prop. of Censoring	25.78	40.42	25.88	40.48

K_U_, K_0.5_, K_W_, and K_BC_, indicates the use of unweighted UniFrac, generalized UniFrac with ϴ = 0.5, weighted UniFrac, and the Bray-Curtis dissimilarity kernels, respectively, for MiRKAT-S [24]

Empirical type I error rates with the proportions of censored outcomes were estimated by setting *β* = (*β*_1_, …, *β*_p_)^′^ = 0. Statistical power was estimated with four different scenarios: (i), 10 most abundant OTUs; (ii), 10 random OTUs; (iii), 10 least abundant OTUs; and (iv), OTUs in a selected cluster performing the partitioning-around-medoids (PAM) algorithm [37], which are associated with survival outcomes, respectively. The first three scenarios evaluate discovery ability when abundant, random/mid-abundant, or rare OTUs are associated. For the fourth scenario, we distributed all OTUs into 10 clusters using the PAM algorithm on the basis of their cophenetic distances in the real phylogenetic tree. The 10 clusters have contained 7.8%, 8.2%, 13.9%, 6.6%, 33.3%, 8.3%, 1.1%, 1.8%, 17.2%, and 1.8% of total reads, respectively, and in each simulation iteration, the selection of one associated cluster was randomized to overcome arbitrary selection. The fourth scenario additionally reflects phylogeny, which may provide a more realistic evaluation.

We also surveyed different effect sizes and directions for the associated OTUs. Λ is denoted as a set of indices for the associated OTUs. For the same effect direction, we set *β*_j ∈ Λ_as a vector of the elements randomly sampled from Unif(0,1), Unif(0,2), or Unif(0,3) and for the mixed effect direction, we set *β*_j ∈ Λ_as a vector of the elements randomly sampled from Unif(− 1,1), Unif(− 2,2), or Unif(− 3,3).

### Simulation results

#### Type I error

We can observe that empirical type I error rates are well-controlled at the significance level of 5% across all the individual and adaptive tests for different censoring schemes and for both small samples (n = 50) and large samples (n = 100) (Table 1).

#### Power

**Fig. 2:**
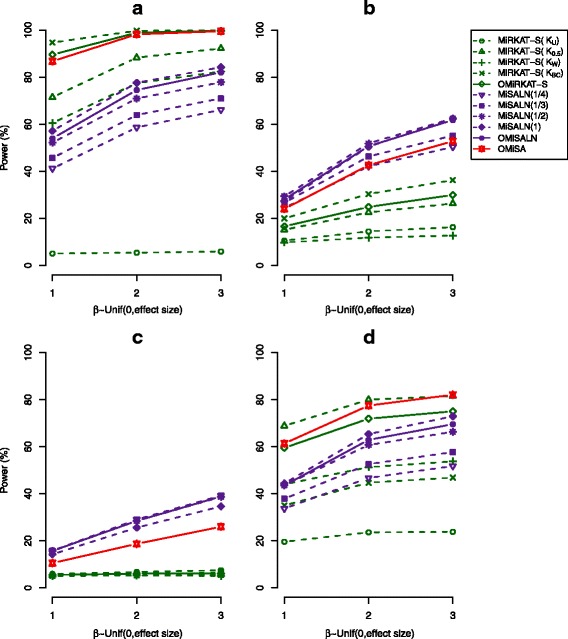
Power estimates for the individual and adaptive tests. The censoring scheme, C_i_ ~ Unif(0,10), and the same effect directions, where *β*_j ∈ Λ_ is a vector of the elements sampled from Unif(0,1) (blue), Unif(0,2) (yellow), or Unif(0,3) (red), for a large sample size (n = 100) were surveyed. K_U_, K_0.5_, K_W_, and K_BC_, indicates the use of unweighted UniFrac, generalized UniFrac with ϴ = 0.5, weighted UniFrac, and the Bray-Curtis dissimilarity kernels, respectively, for MiRKAT-S [24]. (**a** 10 most abundant OTUs are associated. **b** 10 random OTUs are associated. **c** 10 least abundant OTUs are associated. **d** OTUs in a chosen cluster are associated)

**Fig. 3:**
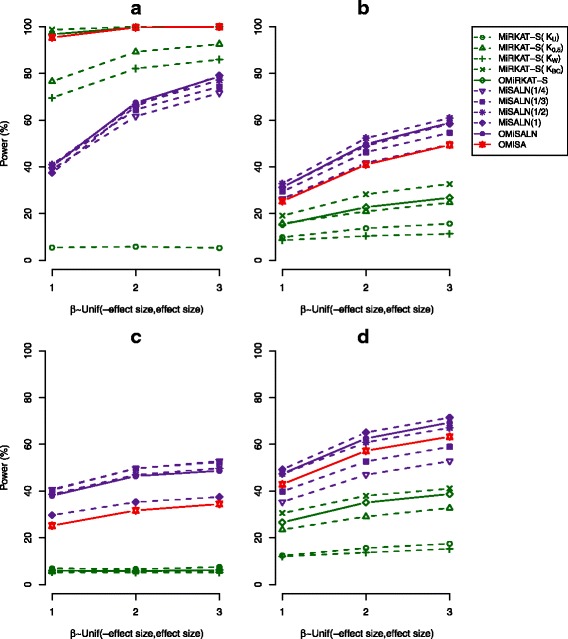
Power estimates for the individual and adaptive tests. The censoring scheme, C_i_ ~ Unif(0,10), and the mixed effect directions, where *β*_j ∈ Λ_ is a vector of the elements sampled from Unif(− 1,1) (blue), Unif(− 2,2) (yellow), or Unif(− 3,3) (red), for a large sample size (n = 100) were surveyed. K_U_, K_0.5_, K_W_, and K_BC_, indicates the use of unweighted UniFrac, generalized UniFrac with ϴ = 0.5, weighted UniFrac, and the Bray-Curtis dissimilarity kernels, respectively, for MiRKAT-S [24]. (**a** 10 most abundant OTUs are associated. **b** 10 random OTUs are associated. **c** 10 least abundant OTUs are associated. **d** OTUs in a chosen cluster are associated)

As we observed similar comparative performances of individual and adaptive tests for different sample sizes and censoring schemes, we include here only the outcomes for large samples (n = 100) and the censoring scheme, Unif(0,10), and moved all of the other outcomes to Additional material (see Additional file 2: Figure S1, Additional file 3: Figure S2, Additional file 4: Figure S3, Additional file 5: Figure S4, Additional file 6: Figure S5, Additional file 7: Figure S6). Figs. 2 and 3 report estimated powers for the same effect direction and mixed effect directions, respectively. We observe that with the increase of effect size, power increases for all the methods under any simulation setting (Figs. 2 and 3), as expected. We also observe that MiRKAT-S/OMiRKAT-S gains slightly more power than MiSALN/OMiSALN for the same effect direction, but it is vice versa for mixed effect directions (Figs. 2 and 3).

MiSALN is powerful using either a large γ value when abundant OTUs are associated with survival outcomes (Figs. 2a and 3a), or using a small γ value when rare OTUs are associated (Figs. 2c and 3c), as expected. For MiRKAT-S, the Bray-Curtis dissimilarity gain power for the first two scenarios in which only the microbial abundances for abundant and random OTUs influence the association (Figs. 2a, b and 3a, b), while the UniFrac distances gain power for the fourth scenario in which phylogenetic tree information is reflected (Figs. 2d and 3d), as expected.

It is notable that the Bray-Curtis dissimilarity is most powerful across all the tests when abundant OTUs are associated with survival outcomes, resulting in high power for OMiRKAT-S (Figs. 2a and 3a). However, as reported in [24], the major problem of MiRKAT-S is that it is underpowered using any distance metric when rare OTUs are associated with survival outcomes; as such, OMiRKAT-S is also underpowered (Figs. 2c and 3c). In contrast, we observed that MiSALN using a small γ value gains power when rare OTUs are associated with survival outcomes, resulting in power for OMiSALN (Figs. 2c and 3c). Overall, we observe that individual item-by-item tests fit specific scenarios, respectively, and there is no single test which fits every scenario. Remarkably, OMiSA is highly robust, approaching the most powerful performance, throughout the four scenarios in which abundant, random, and rare OTUs are associated with survival outcomes and phylogenetic tree information is present (Figs. 2a-d and 3a-d).

### Software comparison

For individual MiRKAT-S tests, we also tried the software package, MiRKATS [24], to determine whether there is any disparity between software facilities. For the use of MiRKATS, we applied the permutation method for small samples (n = 50) and the analytic *p*-value calculation for large samples (n = 100), as suggested [24]. We show that two software packages, OMiSA (our software) and MiRKATS [24], produce nearly identical power estimates for individual MiRKAT-S tests (see Additional file 8: Figure S7, Additional file 9: Figure S8, Additional file 10: Figure S9, Additional file 11: Figure S10, Additional file 12: Figure S11, Additional file 13: Figure S12, Additional file 14: Figure S13, Additional file 15: Figure S14).

### Real data analysis

Early-life interactions between microbiota and their hosts have been considered as potential key factors in immunological and metabolic development [38]. Type 1 diabetes (T1D) is an autoimmune disease which is growing in incidence with decreasing age of onset [39]. Livanos et al. performed a microbiome profiling study to survey whether antibiotic-mediated gut microbiome perturbation accelerates onset of T1D in mice [23]. For the study, NOD mice were exposed to different antibiotic treatments or not, and their intestinal microbial populations were sequenced over time, as described in detail [23]. In brief, fecal, cecal, or ileal specimens from NOD mice were collected and the V4 region of bacterial 16S rRNA gene was amplified by triplicate PCR (F515/R806) using barcoded fusion primers. OTUs, as well as their phylogenetic relationships, were examined using the QIIME pipeline [8].

The data are extensive and motivate diverse study orientations, but, here, we analyze whether perturbed microbial composition by antibiotic use is associated with T1D survival. This analysis is necessary as a part of mediation analysis to understand the process by which the antibiotic use causally affects T1D development [40]. As a demonstration, we used 19 fecal samples from male NOD mice at 6 weeks of age in therapeutic-dose pulsed antibiotic (PAT) treatment. The mice were followed for 30 weeks, during this time, 10 mice developed T1D, while 9 mice did not. Livanos et al. [23] also performed analyses to determine differences in relative abundance of genera between the T1D-free and T1D-development groups at 30 weeks of follow-up. However, those analyses were limited to surveying the binary T1D status (T1D-free vs. T1D-development) at an arbitrary single time point. Thus, we re-analyzed the data by taking the entire survival process into account using our proposed methods. We applied a filtering rule, a proportion mean > 10^−4^, identifying 120 OTUs in the entire community. We first conducted association testing for the entire community (i.e., bacterial kingdom) using the individual and adaptive tests. Then, we tested microbial taxa at five different taxonomic levels, phylum, class, order, family, and genus, respectively. We tested microbial taxa that have ≥2 OTUs in the data using OMiSA and microbial taxa that have only one OTU in the data using univariate Cox proportional hazards models. At each taxonomic level, we omitted OTUs that do not have any taxonomy assignment. For multiple testing correction, we applied the Benjamini-Hochberg (BH) procedure [41] per taxonomic level. However, we are not restricted to the use of BH procedure for multiple testing correction and other less conservative procedures might be considered [42]. No covariate adjustments were included as with [23].

Table 2 reports adjusted *p*-values for the entire community using the individual and adaptive tests. Based on either of the adaptive tests, OMiSALN, OMiRKAT-S, and OMiSA, we observe that the microbial composition of the entire community is significantly associated with T1D survival (Table 2). Here, we further observe that MiRKAT-S using the Bray-Curtis dissimilarity has the smallest *p*-value among all the individual tests and the *p*-value for MiSALN is decreasing as the specified γ value is increasing (Table 2). This may indicate that relatively abundant OTUs in the entire community are associated with T1D survival in microbial abundance.

**Table 2:** The estimated *p*-values for the association between microbial composition of the entire community and T1D survival

Method	*p*-value
MiRKAT-S(K_U_)	0.043
MiRKAT-S(K_0.5_)	0.012
MiRKAT-S(K_W_)	0.009
MiRKAT-S(K_BC_)	0.004
OMiRKAT-S	0.006
MiSALN(1/4)	0.025
MiSALN(1/3)	0.024
MiSALN(1/2)	0.023
MiSALN(1)	0.022
OMiSALN	0.026
OMiSA	0.006

K_U_, K_0.5_, K_W_, and K_BC_, indicates the use of unweighted UniFrac, generalized UniFrac with ϴ = 0.5, weighted UniFrac, and the Bray-Curtis dissimilarity kernels, respectively, for MiRKAT-S [24]

**Fig. 4:**
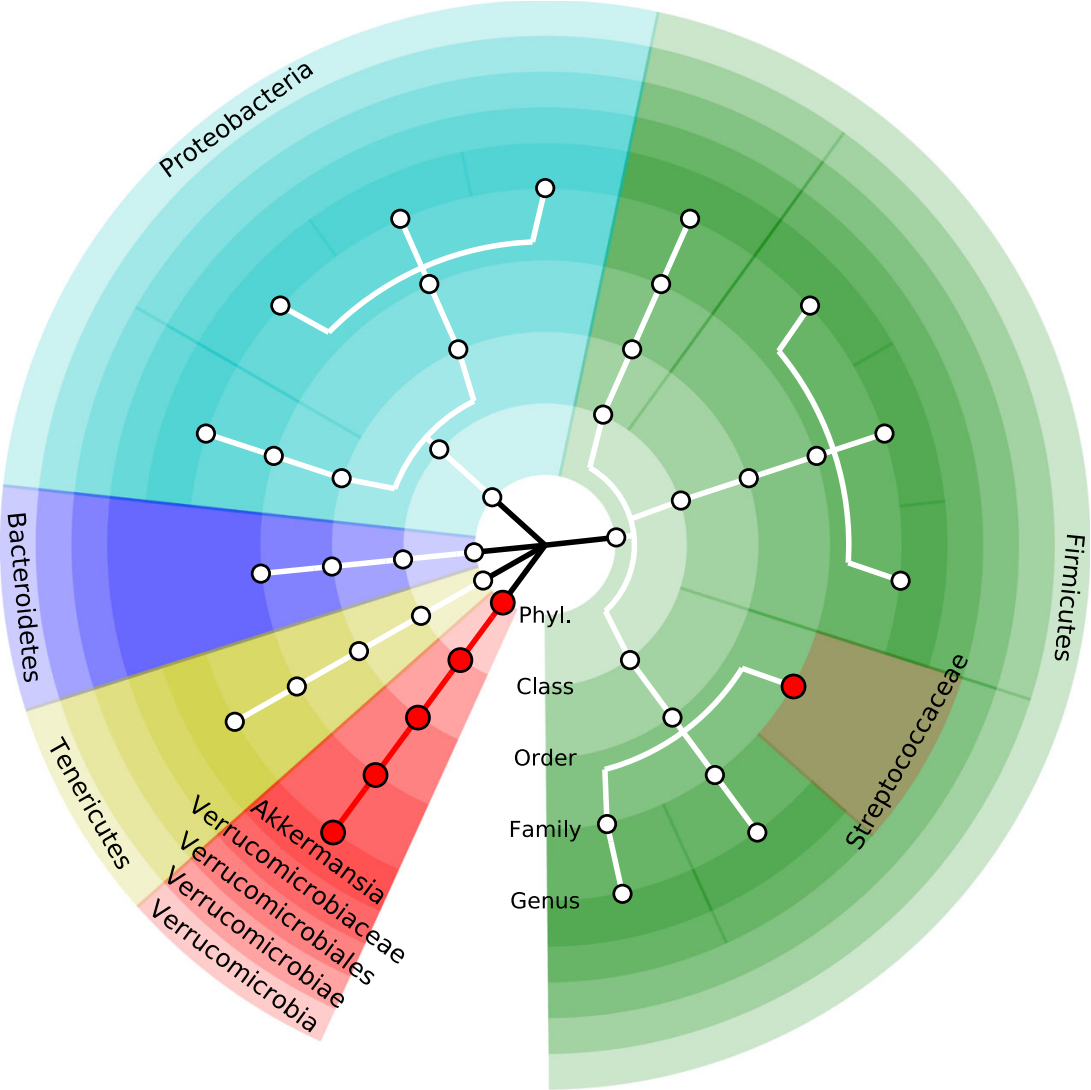
The circular representations of hierarchical taxonomic tree to organize discovered microbial taxa (red circles) at five different taxonomic levels, phylum, class, order, family, and genus. The software tool, GraPhlAn, was used [34]

We created Fig. 4 using a software tool, GraPhlAn, for circular representations of hierarchical taxonomic tree [34] and Fig. 4 summarizes discovered taxa (red circles). We observe that the microbial composition of a phylum, Verrucomicrobia (adj. *p*: 0.005), a class, Verrucomicrobiae (adj. *p*: <.001), an order, Verrucomicrobiales (adj. *p*: <.001), two families, Verrucomicrobiaceae (adj. *p*: <.001) and Streptococcaceae (adj. *p*: 0.040), and a genus, Akkermansia (adj. *p*: 0.012), are significantly associated with T1D survival (Fig. 4).

## Discussion

We described that our computational procedures are efficient as they are based on the score-based tests without double permutations (see Additional file 1), but Plantinga et al.’s analytical *p*-value calculation - based on a closed form asymptotic null distribution suggested for large samples (n > 100) - should be more efficient [24]. Testing all higher-level taxa throughout all different taxonomic ranks may impose a greater computational burden on OMiSA. Thus, for such complete association mapping, we suggest to use multi-core computers to simultaneously implement multiple OMiSA tests. Our software package is currently written in R to exploit existing R functions, but the use of lower-level languages (e.g., C or Fortran) may further enhance its computational performance. Although OMiSA approaches the smallest *p*-value and thus the highest power adaptively, varying *p*-values from individual candidate tests may provide some biological insights on the basis of their methodological aspects and simulation outcomes (Figs. 2 and 3). For MiSALN, we may, in reverse, infer that when the use of a small γ value gains a relatively smaller *p*-value, rare OTUs might be associated, but it is vice versa when the use of a large γ value gains a relatively smaller *p*-value. Similarly, for MiRKAT-S, when the use of UniFrac distances gains a relatively smaller *p*-value than the use of Bray-Curtis dissimilarity, associated OTUs might be phylogenetically related, while when the use of Bray-Curtis dissimilarity gains a relatively smaller *p*-value than the use of UniFrac distances, associated OTUs might not be phylogenetically related. However, “rare or abundant” and “phylogenetically related or not” are conceptual terms with no firm definition, so such interpretations might be challenging.

It is a well-recognized issue in the microbiome research community that the compositional nature of data renders spurious correlation among microbial markers by the unit sum constraint per sample. As a remedy, diverse data transformation procedures (e.g., log-ratio transformation [43]) have been studied, but it is still highly debatable on which procedure is the most robust one [13, 44]. Thus, here, we did not employ any specific data transformation procedure, but would let users decide. For example, centered log-ratio transformation [43] can be considered for OMiSALN as it is usable for either data format in count or composition.

While we illustrated our proposed methods to be used for microbiota profiling studies via 16S rRNA gene target sequencing, they are transferrable to metagenomic studies via whole microbial genome sequencing [45]. As long as the data are organized for a group of multiple count markers with their phylogenetic tree structure, any of our proposed methods can be readily used.

## Conclusions

As current item-by-item approaches have limitations due to the unknown true association pattern, we introduced three new adaptive tests, OMiSALN, OMiRKAT-S, and OMiSA, for microbiome-based association studies with survival outcomes. We ascertained that they are all statistically valid with well-controlled type I error rates for different sample sizes and censoring proportions. Among those, our major proposed method, OMiSA, is highly attractive, as it is robustly powerful through a breadth of association patterns. We also presented that it is not restricted to test the entire community, but also applicable to any taxonomic level above species, and our residual-based permutation method always produces a closed form *p*-value (see Additional file 1 [14, 15, 20, 46]). Consequently, we recommend that OMiSA can extensively be used for microbiome-based survival analysis as a robust group analytic method.

## Additional files

Additional file 1: Computational procedures. (PDF 390 kb)

Additional file 2: **Figure S1.** Power estimates for the individual and adaptive tests. The censoring scheme, C_i_ ~ Unif(0,5), and the same effect directions, where *β*_j ∈ Λ_ is a vector of the elements sampled from Unif(0,1) (blue), Unif(0,2) (yellow), or Unif(0,3) (red), for a large sample size (n = 100) were surveyed. K_U_, K_0.5_, K_W_, and K_BC_, indicates the use of unweighted UniFrac, generalized UniFrac with ϴ = 0.5, weighted UniFrac, and the Bray-Curtis dissimilarity kernels, respectively, for MiRKAT-S [24]. (A: 10 most abundant OTUs are associated. B: 10 random OTUs are associated. C: 10 least abundant OTUs are associated. D: OTUs in a chosen cluster are associated.). (PDF 10 kb)

Additional file 3: **Figure S2.** Power estimates for the individual and adaptive tests. The censoring scheme, C_i_ ~ Unif(0,5), and the mixed effect directions, where *β*_j ∈ Λ_ is a vector of the elements sampled from Unif(− 1,1) (blue), Unif(− 2,2) (yellow), or Unif(− 3,3) (red), for a large sample size (n = 100) were surveyed. K_U_, K_0.5_, K_W_, and K_BC_, indicates the use of unweighted UniFrac, generalized UniFrac with ϴ = 0.5, weighted UniFrac, and the Bray-Curtis dissimilarity kernels, respectively, for MiRKAT-S [24]. (A: 10 most abundant OTUs are associated. B: 10 random OTUs are associated. C: 10 least abundant OTUs are associated. D: OTUs in a chosen cluster are associated.). (PDF 10 kb)

Additional file 4: **Figure S3.** Power estimates for the individual and adaptive tests. The censoring scheme, C_i_ ~ Unif(0,10), and the same effect directions, where *β*_j ∈ Λ_ is a vector of the elements sampled from Unif(0,1) (blue), Unif(0,2) (yellow), or Unif(0,3) (red), for a small sample size (n = 50) were surveyed. K_U_, K_0.5_, K_W_, and K_BC_, indicates the use of unweighted UniFrac, generalized UniFrac with ϴ = 0.5, weighted UniFrac, and the Bray-Curtis dissimilarity kernels, respectively, for MiRKAT-S [24]. (A: 10 most abundant OTUs are associated. B: 10 random OTUs are associated. C: 10 least abundant OTUs are associated. D: OTUs in a chosen cluster are associated.). (PDF 9 kb)

Additional file 5: **Figure S4.** Power estimates for the individual and adaptive tests. The censoring scheme, C_i_ ~ Unif(0,10), and the mixed effect directions, where *β*_j ∈ Λ_ is a vector of the elements sampled from Unif(0,1) (blue), Unif(0,2) (yellow), or Unif(0,3) (red), for a small sample size (n = 50) were surveyed. K_U_, K_0.5_, K_W_, and K_BC_, indicates the use of unweighted UniFrac, generalized UniFrac with ϴ = 0.5, weighted UniFrac, and the Bray-Curtis dissimilarity kernels, respectively, for MiRKAT-S [24]. (A: 10 most abundant OTUs are associated. B: 10 random OTUs are associated. C: 10 least abundant OTUs are associated. D: OTUs in a chosen cluster are associated.). (PDF 9 kb)

Additional file 6: **Figure S5.** Power estimates for the individual and adaptive tests. The censoring scheme, C_i_ ~ Unif(0,5), and the same effect directions, where *β*_j ∈ Λ_ is a vector of the elements sampled from Unif(0,1) (blue), Unif(0,2) (yellow), or Unif(0,3) (red), for a small sample size (n = 50) were surveyed. K_U_, K_0.5_, K_W_, and K_BC_, indicates the use of unweighted UniFrac, generalized UniFrac with ϴ = 0.5, weighted UniFrac, and the Bray-Curtis dissimilarity kernels, respectively, for MiRKAT-S [24]. (A: 10 most abundant OTUs are associated. B: 10 random OTUs are associated. C: 10 least abundant OTUs are associated. D: OTUs in a chosen cluster are associated.). (PDF 9 kb)

Additional file 7: **Figure S6.** Power estimates for the individual and adaptive tests. The censoring scheme, C_i_ ~ Unif(0,5), and the mixed effect directions, where *β*_j ∈ Λ_ is a vector of the elements sampled from Unif(− 1,1) (blue), Unif(− 2,2) (yellow), or Unif(− 3,3) (red), for a small sample size (n = 50) were surveyed. K_U_, K_0.5_, K_W_, and K_BC_, indicates the use of unweighted UniFrac, generalized UniFrac with ϴ = 0.5, weighted UniFrac, and the Bray-Curtis dissimilarity kernels, respectively, for MiRKAT-S [24]. (A: 10 most abundant OTUs are associated. B: 10 random OTUs are associated. C: 10 least abundant OTUs are associated. D: OTUs in a chosen cluster are associated.). (PDF 9 kb)

Additional file 8: **Figure S7.** Power estimates for individual MiRKAT-S tests through different software facilities, OMiSA and MiRKATS (via analytic *p*-value calculation). The censoring scheme, C_i_ ~ Unif(0,10), and the same effect directions, where *β*_j ∈ Λ_ is a vector of the elements sampled from Unif(0,1) (blue), Unif(0,2) (yellow), or Unif(0,3) (red), for a large sample size (n = 100) were surveyed. K_U_, K_0.5_, K_W_, and K_BC_, indicates the use of unweighted UniFrac, generalized UniFrac with ϴ = 0.5, weighted UniFrac, and the Bray-Curtis dissimilarity kernels, respectively. (PDF 6 kb)

Additional file 9: **Figure S8.** Power estimates for individual MiRKAT-S tests through different software facilities, OMiSA and MiRKATS (via analytic *p*-value calculation). The censoring scheme, C_i_ ~ Unif(0,10), and the mixed effect directions, where *β*_j ∈ Λ_ is a vector of the elements sampled from Unif(− 1,1) (blue), Unif(− 2,2) (yellow), or Unif(− 3,3) (red), for a large sample size (n = 100) were surveyed. K_U_, K_0.5_, K_W_, and K_BC_, indicates the use of unweighted UniFrac, generalized UniFrac with ϴ = 0.5, weighted UniFrac, and the Bray-Curtis dissimilarity kernels, respectively. (PDF 6 kb)

Additional file 10: **Figure S9.** Power estimates for individual MiRKAT-S tests through different software facilities, OMiSA and MiRKATS (via analytic *p*-value calculation). The censoring scheme, C_i_ ~ Unif(0,5), and the same effect directions, where *β*_j ∈ Λ_ is a vector of the elements sampled from Unif(0,1) (blue), Unif(0,2) (yellow), or Unif(0,3) (red), for a large sample size (n = 100) were surveyed. K_U_, K_0.5_, K_W_, and K_BC_, indicates the use of unweighted UniFrac, generalized UniFrac with ϴ = 0.5, weighted UniFrac, and the Bray-Curtis dissimilarity kernels, respectively. (PDF 6 kb)

Additional file 11: **Figure S10.** Power estimates for individual MiRKAT-S tests through different software facilities, OMiSA and MiRKATS (via analytic *p*-value calculation). The censoring scheme, C_i_ ~ Unif(0,5), and the mixed effect directions, where *β*_j ∈ Λ_ is a vector of the elements sampled from Unif(− 1,1) (blue), Unif(− 2,2) (yellow), or Unif(− 3,3) (red), for a large sample size (n = 100) were surveyed. K_U_, K_0.5_, K_W_, and K_BC_, indicates the use of unweighted UniFrac, generalized UniFrac with ϴ = 0.5, weighted UniFrac, and the Bray-Curtis dissimilarity kernels, respectively. (PDF 6 kb)

Additional file 12: **Figure S11.** Power estimates for individual MiRKAT-S tests through different software facilities, OMiSA and MiRKATS (via permutation). The censoring scheme, C_i_ ~ Unif(0,10), and the same effect directions, where *β*_j ∈ Λ_ is a vector of the elements sampled from Unif(0,1) (blue), Unif(0,2) (yellow), or Unif(0,3) (red), for a small sample size (n = 50) were surveyed. K_U_, K_0.5_, K_W_, and K_BC_, indicates the use of unweighted UniFrac, generalized UniFrac with ϴ = 0.5, weighted UniFrac, and the Bray-Curtis dissimilarity kernels, respectively. (PDF 6 kb)

Additional file 13: **Figure S12.** Power estimates for individual MiRKAT-S tests through different software facilities, OMiSA and MiRKATS (via permutation). The censoring scheme, C_i_ ~ Unif(0,10), and the mixed effect directions, where *β*_j ∈ Λ_ is a vector of the elements sampled from Unif(− 1,1) (blue), Unif(− 2,2) (yellow), or Unif(− 3,3) (red), for a small sample size (n = 50) were surveyed. K_U_, K_0.5_, K_W_, and K_BC_, indicates the use of unweighted UniFrac, generalized UniFrac with ϴ = 0.5, weighted UniFrac, and the Bray-Curtis dissimilarity kernels, respectively. (PDF 6 kb)

Additional file 14: **Figure S13.** Power estimates for individual MiRKAT-S tests through different software facilities, OMiSA and MiRKATS (via permutation). The censoring scheme, C_i_ ~ Unif(0,5), and the same effect directions, where *β*_j ∈ Λ_ is a vector of the elements sampled from Unif(0,1) (blue), Unif(0,2) (yellow), or Unif(0,3) (red), for a small sample size (n = 50) were surveyed. K_U_, K_0.5_, K_W_, and K_BC_, indicates the use of unweighted UniFrac, generalized UniFrac with ϴ = 0.5, weighted UniFrac, and the Bray-Curtis dissimilarity kernels, respectively. (PDF 6 kb)

Additional file 15: **Figure S14.** Power estimates for individual MiRKAT-S tests through different software facilities, OMiSA and MiRKATS (via permutation). The censoring scheme, C_i_ ~ Unif(0,5), and the mixed effect directions, where *β*_j ∈ Λ_ is a vector of the elements sampled from Unif(− 1,1) (blue), Unif(− 2,2) (yellow), or Unif(− 3,3) (red), for a small sample size (n = 50) were surveyed. K_U_, K_0.5_, K_W_, and K_BC_, indicates the use of unweighted UniFrac, generalized UniFrac with ϴ = 0.5, weighted UniFrac, and the Bray-Curtis dissimilarity kernels, respectively. (PDF 6 kb)

## **Abbreviations**

BH: Benjamini-Hochberg
MiRKAT: Microbiome regression-based kernel association test
MiRKAT-S: Microbiome regression-based kernel association test for survival traits
MiSALN: Microbiome-based survival analysis using linear and non-linear bases of OTUs
NOD: Non-obese diabetic
OMiAT: Optimal microbiome-based association test
OMiRKAT-S: Optimal microbiome regression-based kernel association test for survival traits
OMiSA: Optimal microbiome-based survival analysis
OMiSALN: Optimal microbiome-based survival analysis using linear and non-linear bases of OTUs
OTU: Operational taxonomic unit
PAM: Partitioning-around-medoids
PAT: Pulsed antibiotic
SPU: The sum of powered score tests
T1D: Type 1 diabetes
UniFrac: Unique fraction

## References

[1] Human Microbiome Project Consortium (2012). A framework for human microbiome research. Nature.

[2] Cho I, Yamanishi S, Cox L, Methé BA, Zavadil J, Li K (2012). Antibiotics in early life alter the murine colonic microbiome and adiposity. Nature.

[3] Cox LM, Yamanish S, Sohn J, Alekseyenko AV, Leung JM, Cho I (2013). Altering the intestinal microbiota during a critical developmental window has lasting metabolic consequences. Cell.

[4] Bokulich NA, Chung J, Battaglia T, Henderson N, Jay M, Li H (2016). Antibiotics, birth mode, and diet shape microbiome maturation during early life. Sci Transl Med.

[5] Mahana D, Trent CM, Kurtz ZD, Bokulich NA, Battaglia T, Chung J (2016). Antibiotic perturbation of the murine gut microbiome enhances the adiposity, insulin resistance, and liver disease associated with high-fat diet. Genome Med.

[6] Woese CR, Fox GE, Zablen L, Uchida T, Bonen L, Pechman K (1975). Conservation of primary structure in 16S ribosomal RNA. Nature.

[7] Hamady M, Knight R (2009). Microbial community profiling for human microbiome projects: tools, techniques. Genome Res.

[8] Caporaso JG, Kuczynski J, Stombaugh J, Bittinger K, Bushman FD, Costello EK (2010). QIIME allows analysis of high-throughput community sequencing data. Nat Methods.

[9] Li H (2015). Microbiome, metagenomics, and high-dimensional compositional data analysis. Annu Rev Stat Appl.

[10] Segata N, Izard J, Waldron L, Gevers D, Miropolsky L, Garrett WS (2011). Metagenomic biomarker discovery and explanation. Genome Biol.

[11] Parks DH, Tyson GW, Hugenholtz P, Beiko RG (2014). STAMP: statistical analysis of taxonomic and functional profiles. Bioinformatics.

[12] Love MI, Huber W, Anders S (2014). Moderated estimation of fold change and dispersion for RNA-seq data with DESeq2. Genome Biol.

[13] Paulson JN, Stine OC, Bravo HC, Pop M (2013). Differential abundance analysis for microbial marker-gene surveys. Nat Methods.

[14] Koh H, Blaser MJ, Li H (2017). A powerful microbiome-based association test and a microbial taxa discovery framework for comprehensive association mapping. Microbiome.

[15] Zhao N, Chen J, Carroll IM, Rinqel-Kulka T, Epstein MP, Zhou H (2015). Testing in microbiome-profiling studies with MiRKAT, the microbiome regression-based kernel association test. Am J Hum Genet.

[16] Lozupone CA, Knight R (2005). UniFrac: a new phylogenetic method for comparing microbial communities. Appl Environ Microbiol.

[17] Lozupone CA, Hamady M, Kelley ST, Knight R (2007). Quantitative and qualitative β diversity measures lead to different insights into factors that structure microbial communities. Appl Environ Microbiol.

[18] Chen J, Bittinger K, Charlson ES, Hoffmann C, Lewis J, Wu GD (2012). Associating microbiome composition with environmental covariates using generalized UniFrac distances. Bioinformatics.

[19] Bray JR, Curtis JT (1957). An ordination of upland forest communities of southern Wisconsin. Ecol Monogr.

[20] Pan W, Kim J, Zhang Y, Shen X, Wei P. A powerful and adaptive association test for rare variants. Genetics. 2014;(4):1081–95. 10.1534/genetics.114.165035 PMC4125385 24831820

[21] Han MK, Zhou Y, Murray S, Tayob N, Noth I, Lama VN (2014). Association between lung microbiome and disease progression in IPF: a prospective cohort study. Lancet Respir Med.

[22] Jenq RR, Taur Y, Devlin SM, Ponce DM, Goldberg JD, Ahr KF (2015). Intestinal Blautia is associated with reduced death from graft-versus-host disease. Biol Blood Marrow Transplants.

[23] Livanos AE, Greiner TU, Vangay P, Pathmasiri W, Stewart D, McRitchie S (2016). Antibiotic-mediated gut microbiome perturbation accelerates development of type 1 diabetes in mice. Nat Microbiol.

[24] Plantinga A, Zhan X, Zhao N, Chen J, Jenq RR, Wu MC (2017). MiRKAT-S: a community-level test of association between the microbiota and survival times. Microbiome.

[25] Ward Jr. JH (1963). Hierarchical grouping to optimize an objective function. J Am Stat Assoc.

[26] Cox D (1972). Regression models and life tables (with discussion). J R Stat Soc Series B.

[27] Lin X, Cai T, Wu MC, Zhou Q, Liu G, Christiani DC (2011). Kernel machine SNP-set analysis for censored survival outcomes in genome-wide association studies. Genet Epidemiol.

[28] Chen H, Lumley T, Brody J, Heard-Costa NL, Fox CS, Cupples LA (2014). Sequence kernel association test for survival traits. Genet Epidemiol.

[29] Verweij PJM, Van Houwelingen HC, Stijnen T (1998). A goodness-of-fit test for Cox's proportional hazards model based on martingale residuals. Biometrics.

[30] Goeman JJ, Oosting J, Cleton-Jansen AM, Anninga JK, Van Houwelingen HC (2005). Testing association of a pathway with survival using gene expression data. Bioinformatics.

[31] Goeman JJ, Van De Geer SA, Van Houwelingen HC (2006). Testing against a high dimensional alternative. J R Stat Soc Series B.

[32] Li H, Chen J (2016). Efficient unified rare variant association test by modeling the population genetic distribution in case-control studies. Genet Epidemiol.

[33] Efron B (1977). The efficiency of Cox’s likelihood function for censored data. J Am Stat Assoc.

[34] Morgan XC, Tickle TL, Sokol H, Gevers D, Devaney KL, Ward DV (2012). Dysfunction of the intestinal microbiome in inflammatory bowel disease and treatment. Genome Biol.

[35] Chen J, Li H (1998). Kernel methods for regression analysis of microbiome composition data. Topics in applied statistics: 2012 symposium of the international Chinese statistical association.

[36] Bender R, Augustin T, Blettner M (2005). Generating survival times to simulate cox proportional hazards models. Stat Med.

[37] Reynolds AP, Richard G, De La Iglesia B, Rayward-Smith VJ (2006). Clustering rules: a comparison of partitioning and hierarchical clustering algorithms. J Math Model Algorithms.

[38] Olszak T, An D, Zeissiq S, Vera MP, Richter J, Franke A (2012). Microbial exposure during early life has persistent effects on natural killer T cell function. Science.

[39] Diamond Project Group (2006). Incidence and trends of childhood type 1 diabetes worldwide 1990-1999. Diabetic Med.

[40] Baron RM, Kenny DA (1986). The moderator-mediator variable distinction in social psychological research: conceptual, strategic and statistical considerations. J Pers Soc Psychol.

[41] Benjamini Y, Hochberg Y (1995). Controlling the false discovery rate: a practical and powerful approach to multiple testing. J R Stat Soc Series B.

[42] Sankaran K, Holmes S. structSSI: simultaneous and selective inference for grouped or hierarchically structured data. J Stat Softw. 2014;59(13) 10.18637/jss.v059.i13 PMC4764101 26917999

[43] Aitchison J (1982). The statistical analysis of compositional data. J R Stat Soc B.

[44] O’Hara RB, Kotze DJ (2010). Do not log-transform count data. Methods Ecol Evol.

[45] Tringe SG, Rubin EM (2005). Metagenomics: DNA sequencing of environmental samples. Nat Rev Genet.

[46] Churchill GA, Doerge RW (1994). Empirical threshold values for quantitative trait mapping. Genetics.

