# Supplementary material for: A highly adaptive microbiome-based association test for survival traits
Source: BMC Genomics. 2018 Mar 20;19:210. doi: 10.1186/s12864-018-4599-8 (PMC5859547; doi:10.1186/s12864-018-4599-8)
Supplement: Supplementary file 1 — Computational procedures. (PDF 390 kb) [file 12864_2018_4599_MOESM1_ESM.pdf]

Here, we describe our computational procedures to estimate p-values for individual MiSALN and MiRKAT-S tests and their adaptive tests, OMiSALN, OMiRKAT-S, and OMiSA. A residual-based permutation method based on the score-based tests was used for the p-value calculation. The score-based tests are closed form estimators which do not require the maximum likelihood estimation (MLE) for the effect of microbial composition on survival outcomes; hence, no convergence problem occurs in the iterative algorithm for MLE. Our residual-based permutation scheme does not require any distributional assumption on the effect of microbial composition; hence, it is robustly valid in the non-parametric setting. Moreover, our computational procedures are efficient, as the score-based tests are easy to compute and we do not double permutations for the adaptive tests. We use the following procedures [14, 15, 20, 46]:

1. Fit the null model (Equation 3) and permute martingale residuals:
   1) Fit a null model (Equation 3) and obtain martingale residuals, $MR_i = d_i - \hat{\Lambda}_i$ for i = 1, ..., n.
   2) Permute martingale residuals and denote each vector of the permuted martingale residuals as $MR^{(b)}$, where b is an index for each vector (b $\in$ {1, ..., B}).
2. Calculate the p-value for $U_{MiSALN(\gamma)}$ (Equation 7) denoting it as $P_{MiSALN(\gamma)}$ and the p-value for $U_{MiRKAT-S(k)}$ (Equation 10) denoting it as $P_{MiRKAT-S(k)}$:
   1) Calculate $U_{MiSALN(\gamma)}$ (Equation 7) and $U_{MiRKAT-S(k)}$ (Equation 10) based on the original martingale residuals.
   2) Calculate $U_{MiSALN(\gamma)}$ (Equation 7) and $U_{MiRKAT-S(k)}$ (Equation 10) under the null based on permuted martingale residuals and denote it as $U^{(b)}_{MiSALN(\gamma)}$ and $U^{(b)}_{MiRKAT-S(k)}$ for b = 1,..., B.
   3) Calculate the p-value, $P_{MiSALN(\gamma)}$, as $P_{MiSALN(\gamma)} = \sum_{b=1}^{B}[I(|U^{(b)}_{MiSALN(\gamma)}| \geq |U_{MiSALN(\gamma)}|)]/B$, and the p-value, $P_{MiRKAT-S(k)}$, as $P_{MiRKAT-S(k)} = \sum_{b=1}^{B}[I(|U^{(b)}_{MiRKAT-S(k)}| \geq |U_{MiRKAT-S(k)}|)]/B$, where $I(\cdot)$ is an indicator function.
3. Calculate the p-value for $Q_{OMiSALN}$ (Equation 8) denoting it as $P_{OMiSALN}$ and the p-value for $Q_{OMiRKAT-S}$ (Equation 11) denoting it as $P_{OMiRKAT-S}$:
   1) Calculate $Q_{OMiSALN}$ (Equation 8) based on the p-values of $P_{MiSALN(\gamma)}$, where $\gamma \epsilon \Gamma$, and $Q_{OMiRKAT-S}$ (Equation 11) based on the p-values of $P_{MiRKAT-S(k)}$, where $k \epsilon \Psi$.
   2) Calculate $Q_{OMiSALN}$ (Equation 8) under the null as $Q^{(b)}_{OMiSALN} = \min_{\gamma \epsilon \Gamma}\{\sum_{b1 \neq b}[I(U^{(b1)}_{MiSALN(\gamma)} \geq U^{(b)}_{MiSALN(\gamma)}) + 1]/(B-1)\}$ and $Q_{OMiRKAT-S}$ (Equation 11) under the null as $Q^{(b)}_{OMiRKAT-S} = \min_{k \epsilon \Psi}\{\sum_{b1 \neq b}[I(U^{(b1)}_{MiRKAT-S(k)} \geq U^{(b)}_{MiRKAT-S(k)}) + 1]/(B-1)\}$, where b and b1 are an index for the same vector of permuted martingale residuals (b $\in$ {1, ..., B}, b1 $\in$ {1, ..., B}).
   3) Calculate the p-value, $P_{OMiSALN}$, as $P_{OMiSALN} = \sum_{b=1}^{B}[I(Q^{(b)}_{OMiSALN} \leq Q_{OMiSALN})]/B$ and the p-value, $P_{OMiRKAT-S}$, as $P_{OMiRKAT-S} = \sum_{b=1}^{B}[I(Q^{(b)}_{OMiRKAT-S} \leq Q_{OMiRKAT-S})]/B$.
4. Calculate the P-value for Q_{OMiSA} (Equation 12) denoting it as P_{OMiSA}:
   1) Calculate Q_{OMiSA} (Equation 12) based on the values of $Q_{OMiSALN}$ and $Q_{OMiRKAT-S}$.
   2) Calculate Q_{OMiSA} (Equation 12) under the null as $Q^{(b)}_{OMiSA} = \min(Q^{(b)}_{OMiSALN}, Q^{(b)}_{OMiRKAT-S})$ for b = 1,..., B.
   3) Calculate the p-value, P_{OMiSA}, as $P_{OMiSA} = \sum_{b=1}^{B}[I(Q^{(b)}_{OMiSA} \leq Q_{OMiSA})]/B$.
