# Supplementary figures and images for: A highly adaptive microbiome-based association test for survival traits
Source: BMC Genomics. 2018 Mar 20;19:210. doi: 10.1186/s12864-018-4599-8 (PMC5859547; doi:10.1186/s12864-018-4599-8)

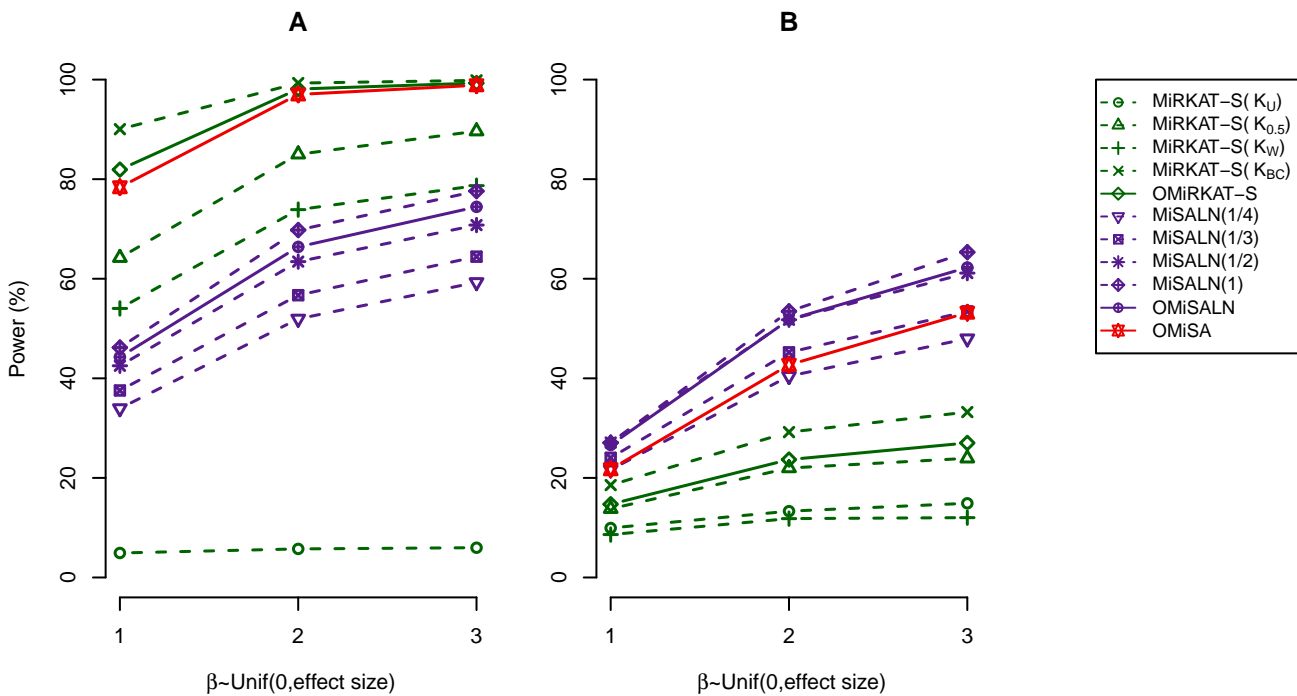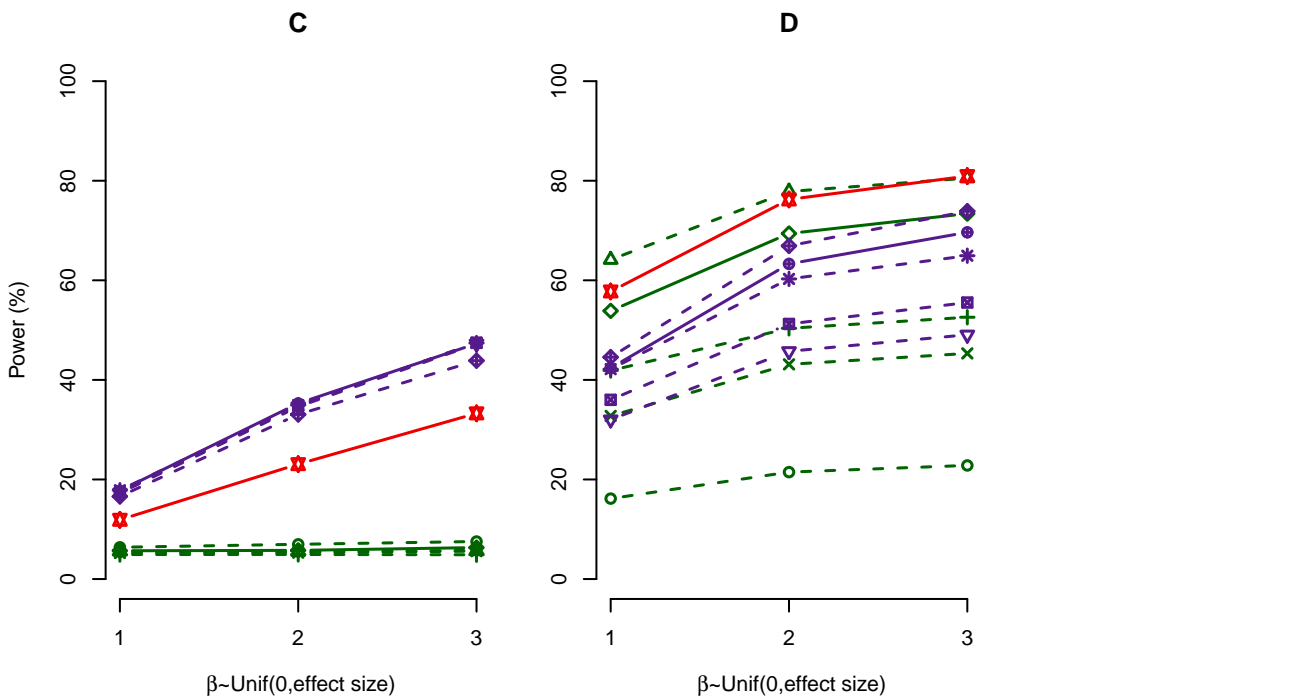

Supplement: Supplementary file 2 — Figure S1. Power estimates for the individual and adaptive tests. The censoring scheme, Ci ~ Unif(0,5), and the same effect directions, where βj ∈ Λ is a vector of the elements sampled from Unif(0,1) (blue), Unif(0,2) (yellow), or Unif(0,3) (red), for a large sample size (n = 100) were surveyed. KU, K0.5, KW, and KBC, indicates the use of unweighted UniFrac, generalized UniFrac with ϴ = 0.5, weighted UniFrac, and the Bray-Curtis dissimilarity kernels, respectively, for MiRKAT-S [24]. (A: 10 most abundant OTUs are associated. B: 10 random OTUs are associated. C: 10 least abundant OTUs are associated. D: OTUs in a chosen cluster are associated.). (PDF 10 kb) [file 12864_2018_4599_MOESM2_ESM.pdf]

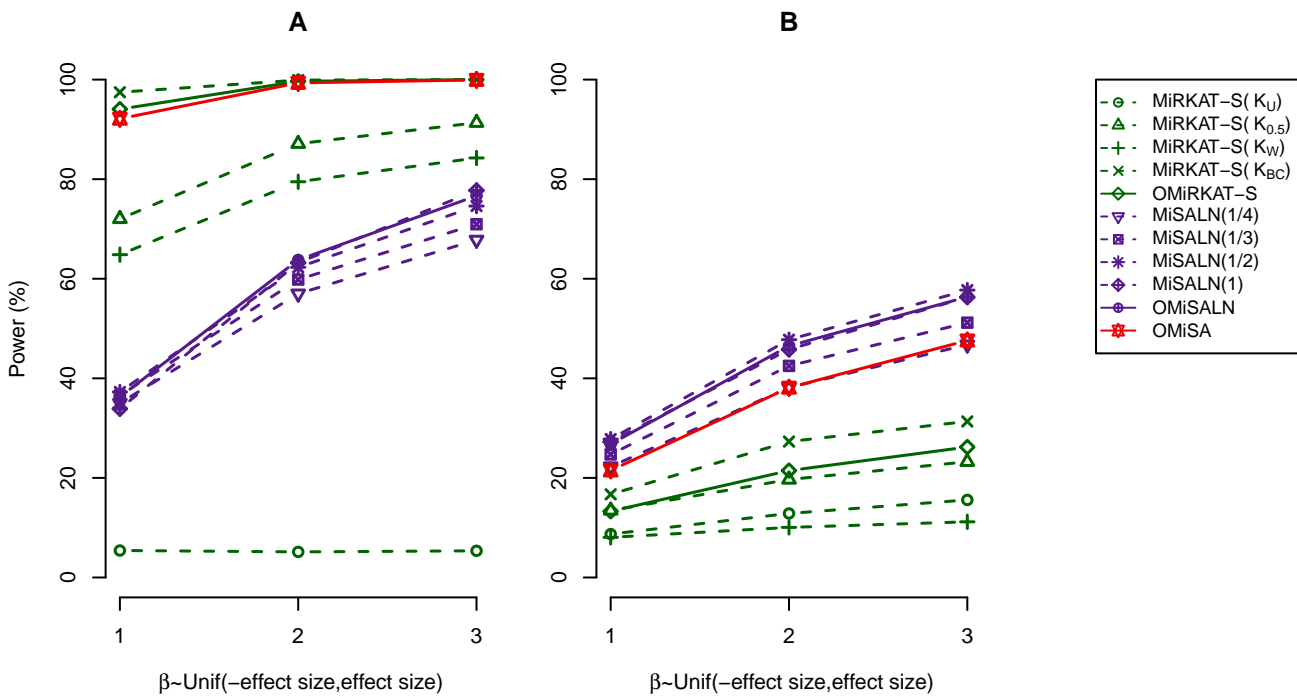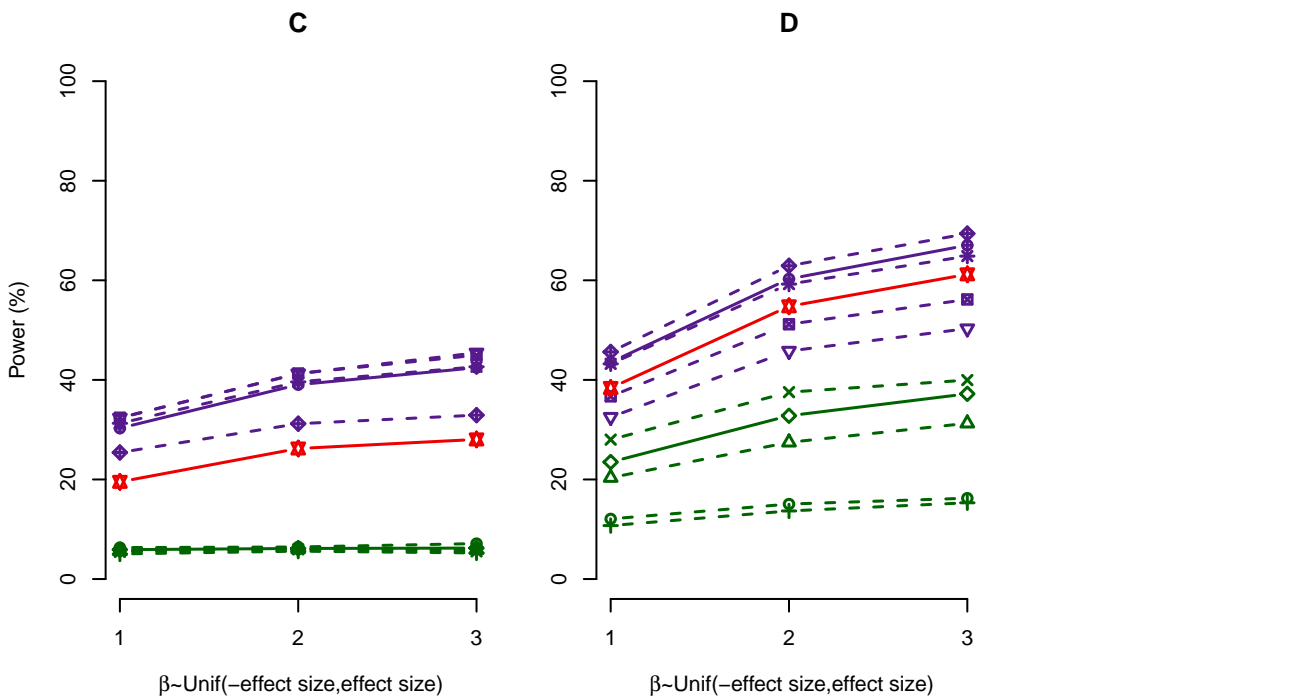

Supplement: Supplementary file 3 — Figure S2. Power estimates for the individual and adaptive tests. The censoring scheme, Ci ~ Unif(0,5), and the mixed effect directions, where βj ∈ Λ is a vector of the elements sampled from Unif(− 1,1) (blue), Unif(− 2,2) (yellow), or Unif(− 3,3) (red), for a large sample size (n = 100) were surveyed. KU, K0.5, KW, and KBC, indicates the use of unweighted UniFrac, generalized UniFrac with ϴ = 0.5, weighted UniFrac, and the Bray-Curtis dissimilarity kernels, respectively, for MiRKAT-S [24]. (A: 10 most abundant OTUs are associated. B: 10 random OTUs are associated. C: 10 least abundant OTUs are associated. D: OTUs in a chosen cluster are associated.). (PDF 10 kb) [file 12864_2018_4599_MOESM3_ESM.pdf]

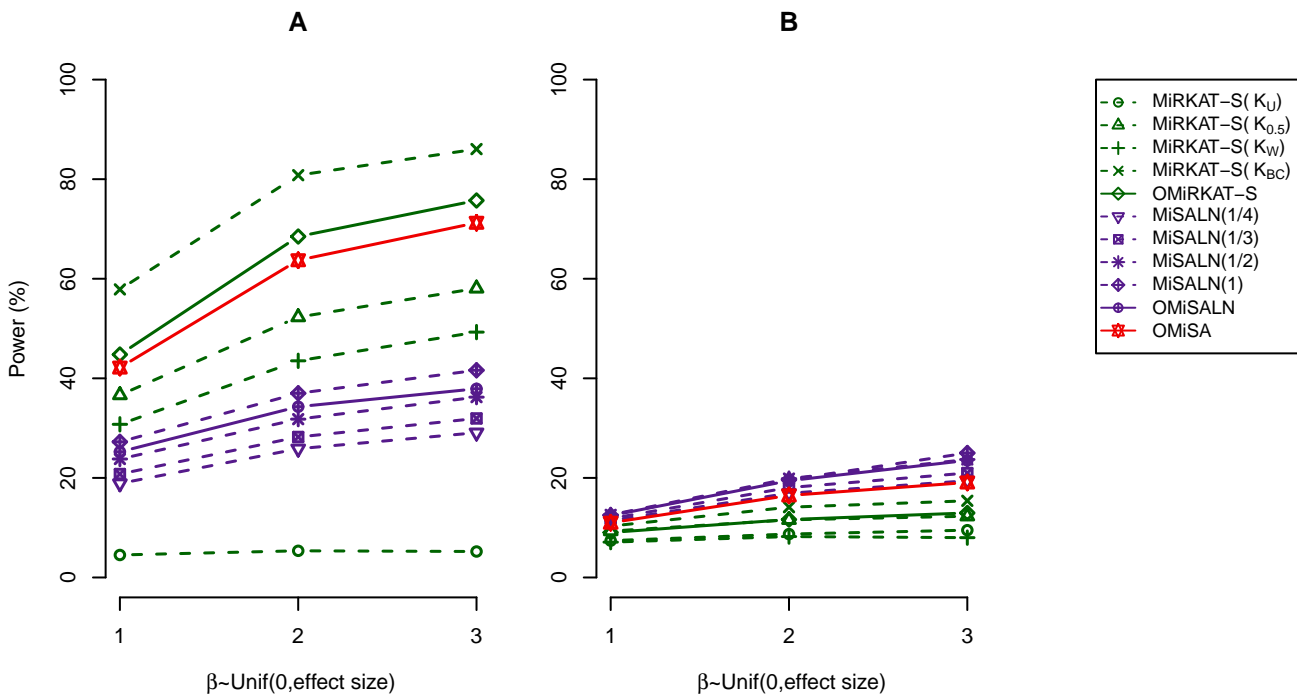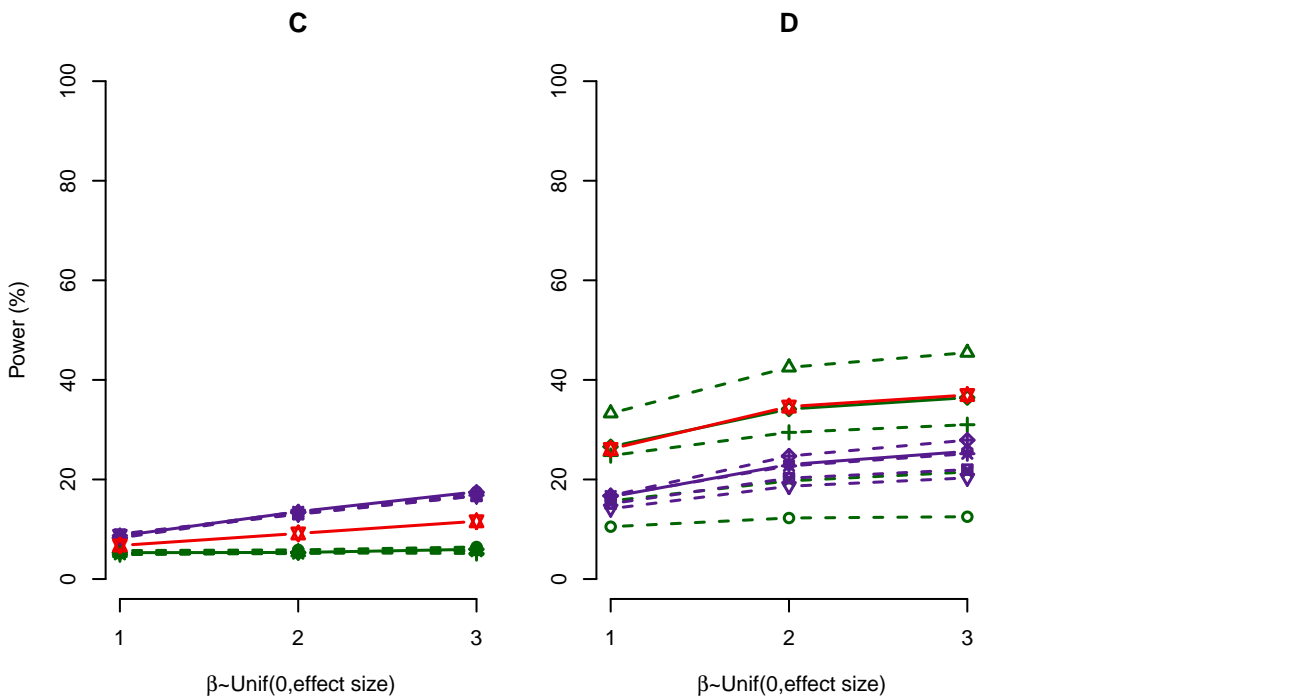

Supplement: Supplementary file 4 — Figure S3. Power estimates for the individual and adaptive tests. The censoring scheme, Ci ~ Unif(0,10), and the same effect directions, where βj ∈ Λ is a vector of the elements sampled from Unif(0,1) (blue), Unif(0,2) (yellow), or Unif(0,3) (red), for a small sample size (n = 50) were surveyed. KU, K0.5, KW, and KBC, indicates the use of unweighted UniFrac, generalized UniFrac with ϴ = 0.5, weighted UniFrac, and the Bray-Curtis dissimilarity kernels, respectively, for MiRKAT-S [24]. (A: 10 most abundant OTUs are associated. B: 10 random OTUs are associated. C: 10 least abundant OTUs are associated. D: OTUs in a chosen cluster are associated.). (PDF 9 kb) [file 12864_2018_4599_MOESM4_ESM.pdf]

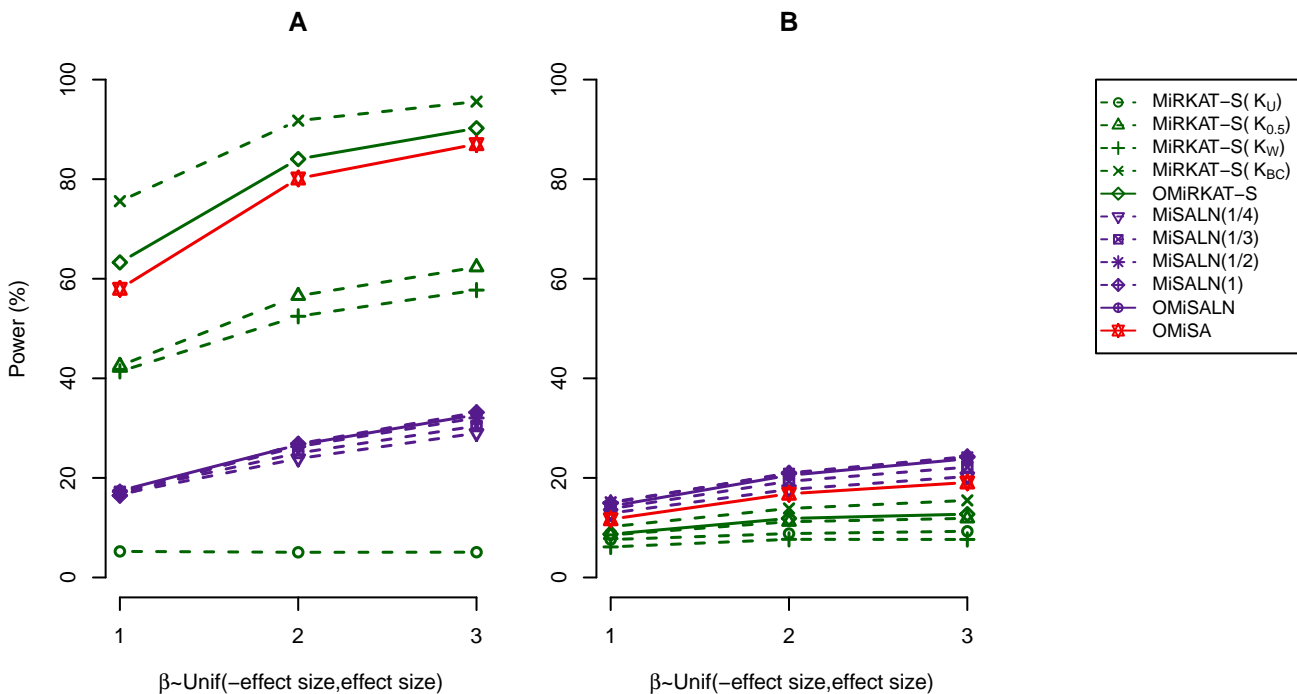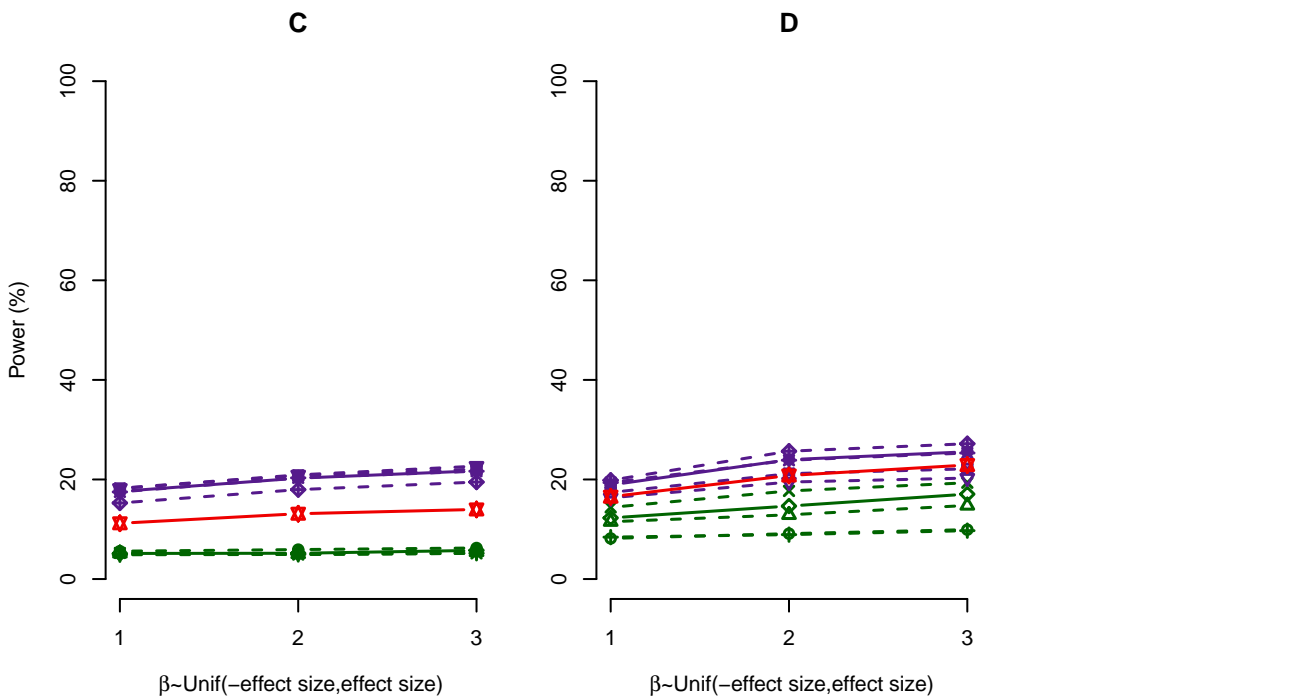

Supplement: Supplementary file 5 — Figure S4. Power estimates for the individual and adaptive tests. The censoring scheme, Ci ~ Unif(0,10), and the mixed effect directions, where βj ∈ Λ is a vector of the elements sampled from Unif(0,1) (blue), Unif(0,2) (yellow), or Unif(0,3) (red), for a small sample size (n = 50) were surveyed. KU, K0.5, KW, and KBC, indicates the use of unweighted UniFrac, generalized UniFrac with ϴ = 0.5, weighted UniFrac, and the Bray-Curtis dissimilarity kernels, respectively, for MiRKAT-S [24]. (A: 10 most abundant OTUs are associated. B: 10 random OTUs are associated. C: 10 least abundant OTUs are associated. D: OTUs in a chosen cluster are associated.). (PDF 9 kb) [file 12864_2018_4599_MOESM5_ESM.pdf]

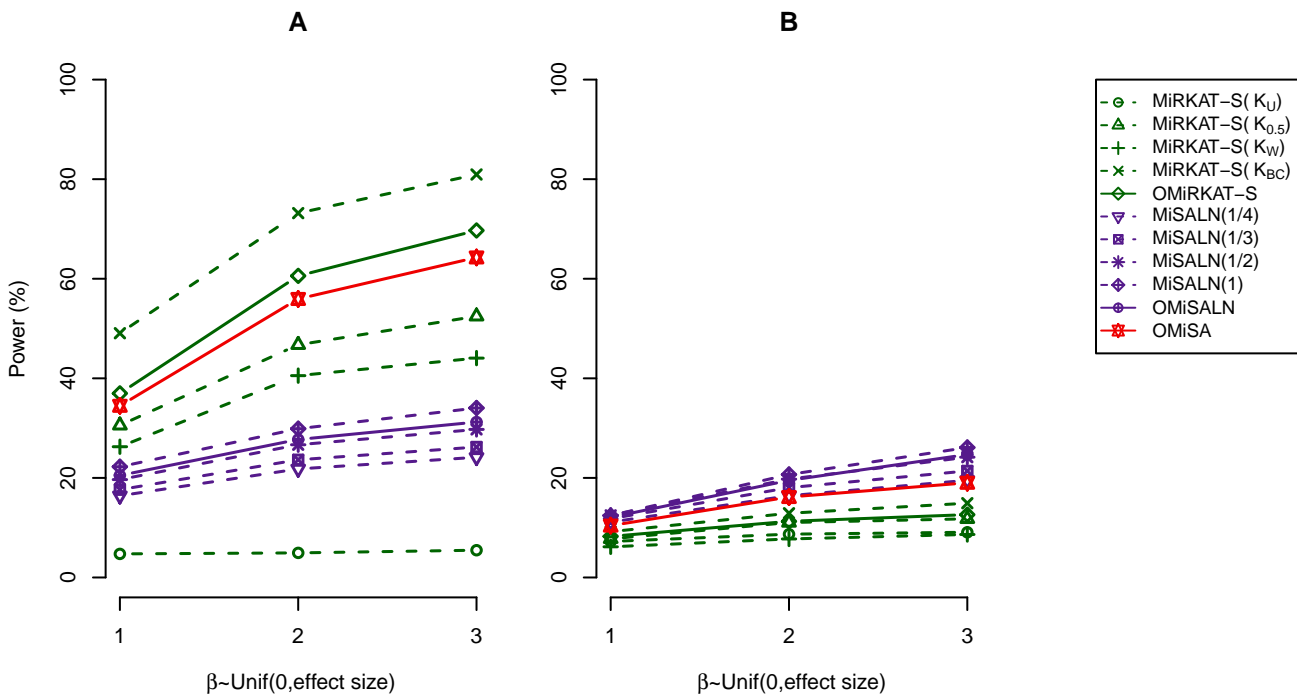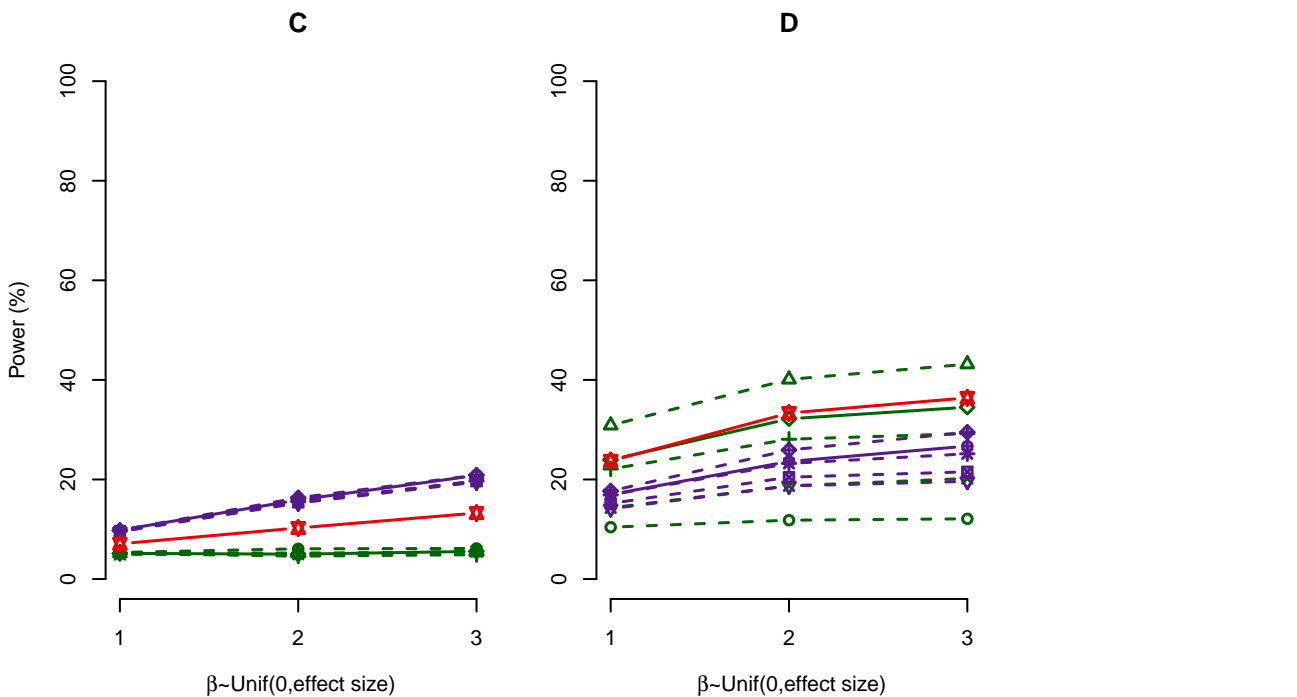

Supplement: Supplementary file 6 — Figure S5. Power estimates for the individual and adaptive tests. The censoring scheme, Ci ~ Unif(0,5), and the same effect directions, where βj ∈ Λ is a vector of the elements sampled from Unif(0,1) (blue), Unif(0,2) (yellow), or Unif(0,3) (red), for a small sample size (n = 50) were surveyed. KU, K0.5, KW, and KBC, indicates the use of unweighted UniFrac, generalized UniFrac with ϴ = 0.5, weighted UniFrac, and the Bray-Curtis dissimilarity kernels, respectively, for MiRKAT-S [24]. (A: 10 most abundant OTUs are associated. B: 10 random OTUs are associated. C: 10 least abundant OTUs are associated. D: OTUs in a chosen cluster are associated.). (PDF 9 kb) [file 12864_2018_4599_MOESM6_ESM.pdf]

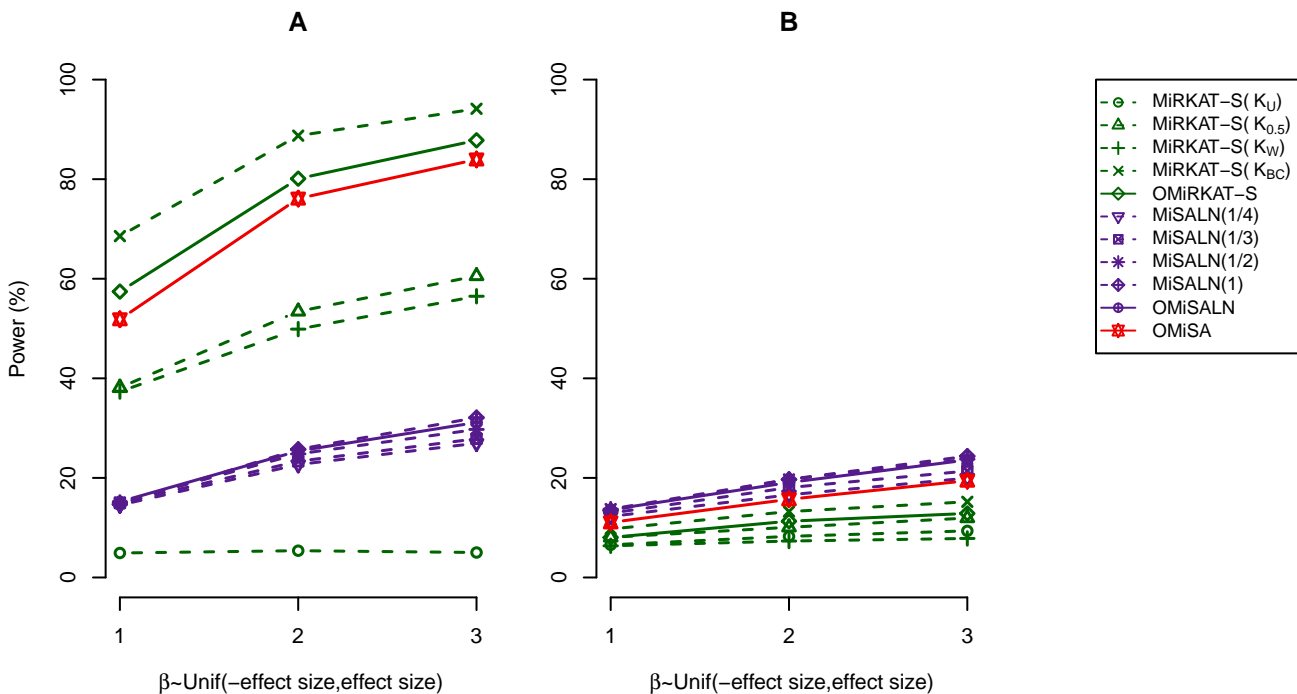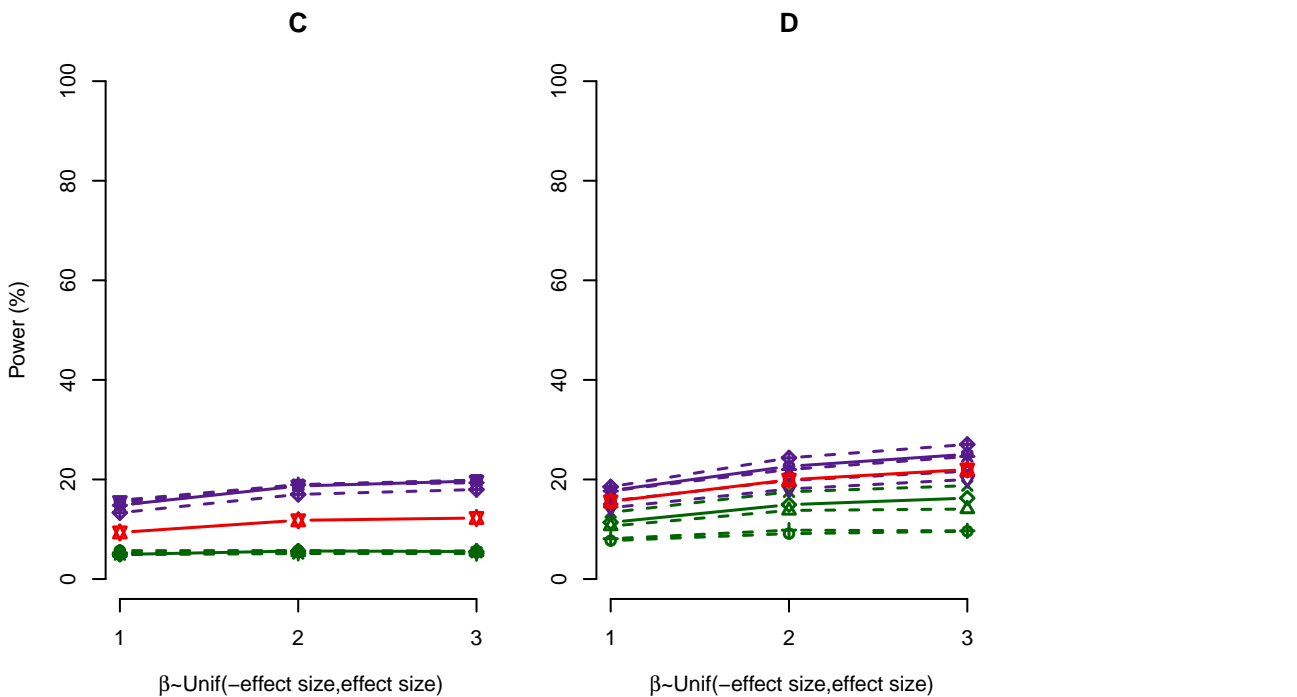

Supplement: Supplementary file 7 — Figure S6. Power estimates for the individual and adaptive tests. The censoring scheme, Ci ~ Unif(0,5), and the mixed effect directions, where βj ∈ Λ is a vector of the elements sampled from Unif(− 1,1) (blue), Unif(− 2,2) (yellow), or Unif(− 3,3) (red), for a small sample size (n = 50) were surveyed. KU, K0.5, KW, and KBC, indicates the use of unweighted UniFrac, generalized UniFrac with ϴ = 0.5, weighted UniFrac, and the Bray-Curtis dissimilarity kernels, respectively, for MiRKAT-S [24]. (A: 10 most abundant OTUs are associated. B: 10 random OTUs are associated. C: 10 least abundant OTUs are associated. D: OTUs in a chosen cluster are associated.). (PDF 9 kb) [file 12864_2018_4599_MOESM7_ESM.pdf]
