# Supplementary material for: A highly adaptive microbiome-based association test for survival traits
Source: BMC Genomics. 2018 Mar 20;19:210. doi: 10.1186/s12864-018-4599-8 (PMC5859547; doi:10.1186/s12864-018-4599-8)
Supplement: Supplementary file 10 — Figure S9. Power estimates for individual MiRKAT-S tests through different software facilities, OMiSA and MiRKATS (via analytic p-value calculation). The censoring scheme, Ci ~ Unif(0,5), and the same effect directions, where βj ∈ Λ is a vector of the elements sampled from Unif(0,1) (blue), Unif(0,2) (yellow), or Unif(0,3) (red), for a large sample size (n = 100) were surveyed. KU, K0.5, KW, and KBC, indicates the use of unweighted UniFrac, generalized UniFrac with ϴ = 0.5, weighted UniFrac, and the Bray-Curtis dissimilarity kernels, respectively. (PDF 6 kb) [file 12864_2018_4599_MOESM10_ESM.pdf]

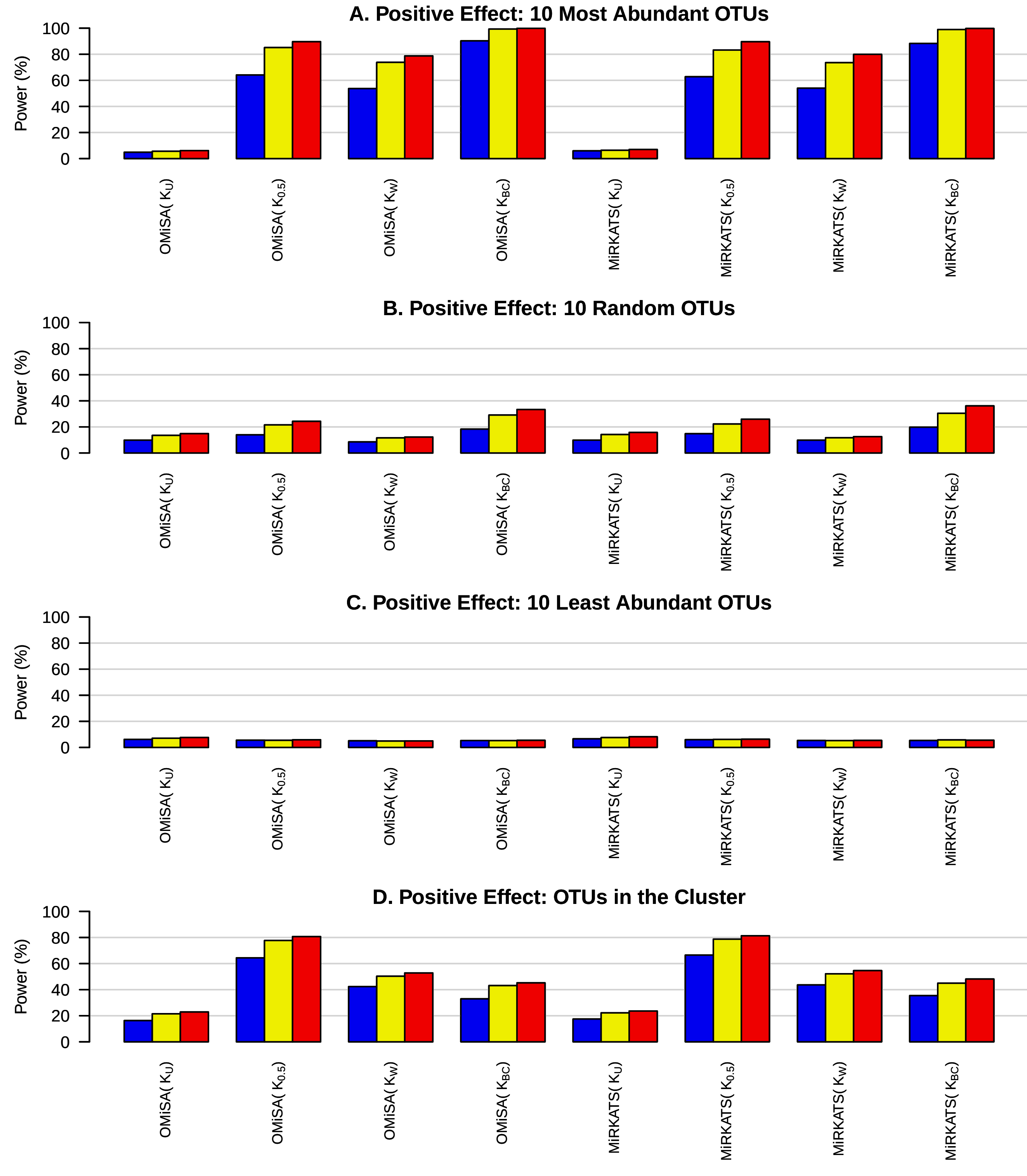

A. Positive Effect: 10 Most Abundant OTUs
B. Positive Effect: 10 Random OTUs
C. Positive Effect: 10 Least Abundant OTUs
D. Positive Effect: OTUs in the Cluster
Power (%)
0
20
40
60
80
100
OMiSA(K_U)
OMiSA(K_0.5)
OMiSA(K_W)
OMiSA(K_BC)
MiRKATS(K_U)
MiRKATS(K_0.5)
MiRKATS(K_W)
MiRKATS(K_BC)
