# Supplementary material for: A highly adaptive microbiome-based association test for survival traits
Source: BMC Genomics. 2018 Mar 20;19:210. doi: 10.1186/s12864-018-4599-8 (PMC5859547; doi:10.1186/s12864-018-4599-8)
Supplement: Supplementary file 13 — Figure S12. Power estimates for individual MiRKAT-S tests through different software facilities, OMiSA and MiRKATS (via permutation). The censoring scheme, Ci ~ Unif(0,10), and the mixed effect directions, where βj ∈ Λ is a vector of the elements sampled from Unif(− 1,1) (blue), Unif(− 2,2) (yellow), or Unif(− 3,3) (red), for a small sample size (n = 50) were surveyed. KU, K0.5, KW, and KBC, indicates the use of unweighted UniFrac, generalized UniFrac with ϴ = 0.5, weighted UniFrac, and the Bray-Curtis dissimilarity kernels, respectively. (PDF 6 kb) [file 12864_2018_4599_MOESM13_ESM.pdf]

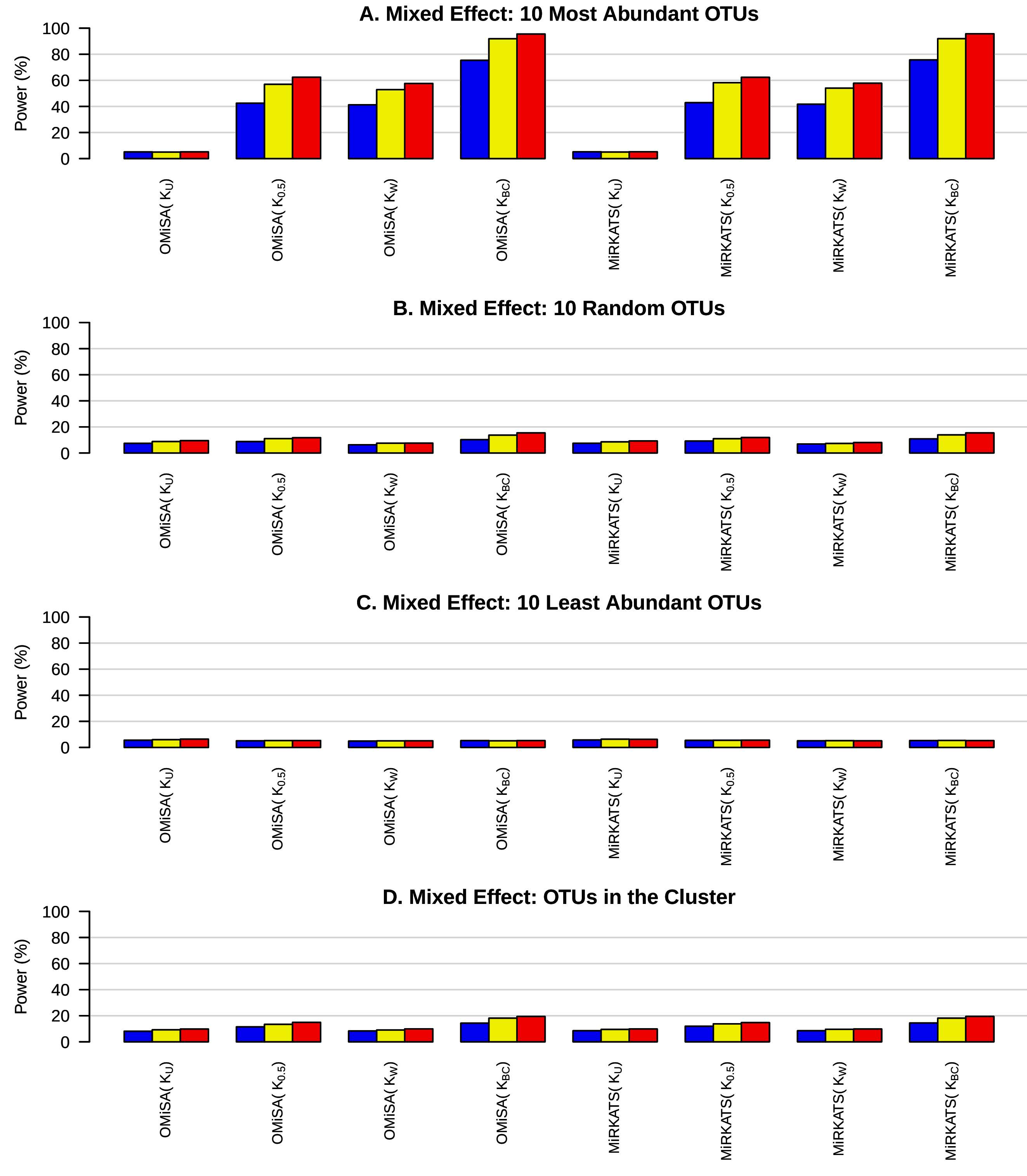
A. Mixed Effect: 10 Most Abundant OTUs
B. Mixed Effect: 10 Random OTUs
C. Mixed Effect: 10 Least Abundant OTUs
D. Mixed Effect: OTUs in the Cluster
Power (%)
0
20
40
60
80
100
OMiSA(KU)
OMiSA(K0.5)
OMiSA(KW)
OMiSA(KBC)
MiRKATS(KU)
MiRKATS(K0.5)
MiRKATS(KW)
MiRKATS(KBC)
